# Physicochemical Characterization, Binder Rheology, and Engineering Performance of Diatomite–Microfiber Hybrid Asphalt Systems

**DOI:** 10.3390/polym18202597

**Published:** 2026-10-21

**Authors:** Mustafa Taha Aslan, Erol İskender

**Affiliations:** 1 Graduate School of Natural and Applied Sciences, Karadeniz Technical University, Trabzon 61080, Türkiye; 2 Department of Civil Engineering, Faculty of Technology, Karadeniz Technical University, Trabzon 61830, Türkiye; eiskender@ktu.edu.tr

**Keywords:** binder rheology, diatomite, fracture performance, hybrid asphalt modification, microfiber reinforcement, physicochemical characterization

## Abstract

Diatomite and microfibers have been investigated as asphalt modifiers, but limited attention has been given to how differences between diatomite sources and microfiber formulations are reflected across binder- and mixture-scale performance. This study investigated two diatomites (D1 and D2) and three microfibers—polyacrylonitrile (PAN), polyethylene terephthalate (PET), and basalt—using a multiscale experimental approach. The diatomites were characterized by particle-size analysis, SEM–EDS, XRF, and XRD, while the fibers were examined using SEM–EDS, XRD, and binder-retention testing. Diatomite-modified binders containing 10% and 15% modifier were evaluated for conventional, rheological, and aging properties. At the mixture scale, selected 15%D1 and 10%D2 systems were combined with the three fibers and compared with unmodified and 5% SBS-modified references. D1 was finer and had a slightly higher SiO_2_ content than D2, while both showed silica-rich porous structures. Diatomite generally increased binder consistency and viscosity and improved the high-temperature rheological response without changing the BBR critical temperature. The 10%D2-PAN mixture achieved the highest indirect tensile strength (4.29 MPa), while the tensile strength ratios of 10%D2-PAN and 10%D2-PET were 82–83%. The 10%D2 formulations showed Cantabro losses of 5.5–6.7% and permanent deformations of 0.247–0.255 mm. In the adapted 100 mm SCB test, 10%D2-PAN also produced the highest flexibility index (10.6) and fracture energy (7.3 kJ/m^2^). Overall, the results indicate that diatomite source characteristics and microfiber formulation should be considered together, with 10%D2-PAN exhibiting a favorable combination of strength, moisture-resistance, particle-loss, permanent-deformation, and fracture-related responses under the investigated conditions.

## 1. Introduction

Asphalt mixtures are multiphase composites whose mechanical response depends on temperature, loading conditions, and interactions among the binder, aggregate, and mineral-filler phases [1]. Increasing traffic demand and environmental exposure subject asphalt pavements to multiple deterioration mechanisms, including permanent deformation at high temperatures, moisture-induced damage, and cracking at low and intermediate temperatures [1,2,3]. At high temperatures, the increased viscous response of the binder can promote irreversible deformation under repeated traffic loading, whereas water infiltration may weaken binder–aggregate adhesion and reduce mixture integrity [2]. At lower temperatures, thermal contraction and reduced stress-relaxation capacity increase the risk of cracking [3]. Because these mechanisms may occur concurrently, improving overall pavement performance through a modification strategy targeting only one response remains challenging.

Polymer modification is widely used to improve asphalt-binder rheology and mixture performance. Styrene–butadiene–styrene (SBS), in particular, can enhance the rheological and mechanical response of asphalt binders; however, its effectiveness depends on factors such as the characteristics of the base binder, SBS structure, modifier content, and aging condition [4]. Pipintakos et al. [4] comparatively evaluated different base binders, SBS structures, and modification levels and demonstrated that both base-binder composition and SBS content substantially influence the physical and chemo-rheological response of polymer-modified binders. More recently, Li et al. [5] investigated reaction-based rejuvenation of aged SBS-modified asphalt and showed that reactive chemicals can mitigate the deterioration in high-temperature performance associated with conventional oil-based rejuvenation. These developments illustrate the continuing effort to optimize the rheological response, aging behavior, and serviceability of SBS-modified binders. Nevertheless, polymer modification may involve limitations related to cost, storage stability, and aging susceptibility [6], motivating continued investigation of mineral modifiers and fibers as alternative or complementary modification approaches.

Among mineral modifiers, diatomite has received attention because of its silica-rich composition and highly porous morphology. Tang et al. [7] investigated the low-temperature behavior of diatomite-modified asphalt mixtures and demonstrated that diatomite modification affected their thermal and mechanical characteristics. Cong et al. [8] subsequently examined the long-term aging behavior of diatomite-containing asphalt binders and reported improved resistance to aging, relating this response to the characteristics of the diatomite-containing system. Changes in asphalt-mixture performance with diatomite content have also been reported [9]. Collectively, these findings indicate that diatomite can influence binder and mixture behavior; however, its effects should not be generalized independently of material source and dosage. Natural diatomites can differ in particle-size distribution, pore structure, surface morphology, elemental composition, and amorphous or crystalline phases, and these source-dependent characteristics may influence their interaction with asphalt binder. Complementary techniques such as SEM–EDS, XRF, and XRD are therefore valuable for distinguishing physicochemical differences between diatomites, although they do not directly quantify surface area, binder absorption, or interfacial adhesion.

Fiber reinforcement provides a complementary approach by introducing mechanisms such as binder stabilization, local stress redistribution, crack bridging, and restriction of crack propagation [10]. The effectiveness of these mechanisms depends on fiber mechanical properties, geometry, surface characteristics, dosage, dispersion, and interaction with the surrounding mastic [10,11]. Basalt fibers provide inorganic reinforcement with relatively high tensile strength and elastic modulus and have been associated with improvements in mixture mechanical and durability-related performance [11,12]. PAN and PET fibers provide chemically distinct polymeric reinforcement systems. Shi et al. [13] investigated the PAN–asphalt interface and demonstrated that the molecular structure of PAN influences its interfacial adhesion characteristics with asphalt, emphasizing the importance of fiber–binder interaction in addition to macroscopic reinforcement. PET fibers have also been investigated as asphalt reinforcement, with improvements in mixture performance reported when recycled PET fibers were incorporated into asphalt concrete [14]. PET additionally offers resource-recovery potential because it can be obtained from recyclable polymer streams. PAN, PET, and basalt therefore represent reinforcing materials with substantially different chemical compositions, mechanical characteristics, surface morphologies, and potential interactions with asphalt mastic.

Research has also progressed from individual mineral or fiber modification toward hybrid systems. Davar et al. [15] investigated combined basalt-fiber and diatomite modification and reported improvements in low-temperature tensile and fatigue-related performance under the investigated conditions. Cheng et al. [11] compared control, diatomite-modified, basalt-fiber-modified, and combined diatomite–basalt-fiber mixtures through high- and low-temperature, moisture, fatigue, and freeze–thaw evaluations, demonstrating complementary contributions of the two modifiers to mixture performance. These studies show that hybrid mineral–fiber modification itself is not a new concept. However, previous hybrid investigations have predominantly focused on specific diatomite–fiber combinations, particularly basalt fiber, and on mixture-level performance. Comparatively less attention has been given to how source-dependent physicochemical differences between diatomites relate to binder rheology and aging and subsequently to the performance of hybrid formulations containing chemically and mechanically distinct microfibers. Integrated studies combining mineral and fiber characterization, binder-scale rheology, fiber–binder retention, mixture microstructure, and multiple mechanical-performance indicators within a common experimental framework remain limited.

The novelty of this study lies not in the individual use of diatomite or fiber modification, which has been previously reported, but in their integrated multiscale evaluation within a common experimental framework. Accordingly, two physicochemically distinct diatomites (D1 and D2) were comparatively characterized and evaluated at the binder scale, while selected diatomite systems were combined with three chemically and mechanically different microfibers (PAN, PET, and basalt) at the mixture scale. The experimental framework integrates modifier characterization, binder rheology and aging, fiber–binder retention, mixture microstructure, and multiple engineering performance indicators to relate source- and formulation-dependent material characteristics to binder- and mixture-scale responses. Because diatomite source and content and fiber type and dosage were not varied independently at the mixture scale, the analysis focuses on the comparative performance of the complete hybrid formulations rather than isolated factor effects.

## 2. Materials and Methods

**Figure 1:**
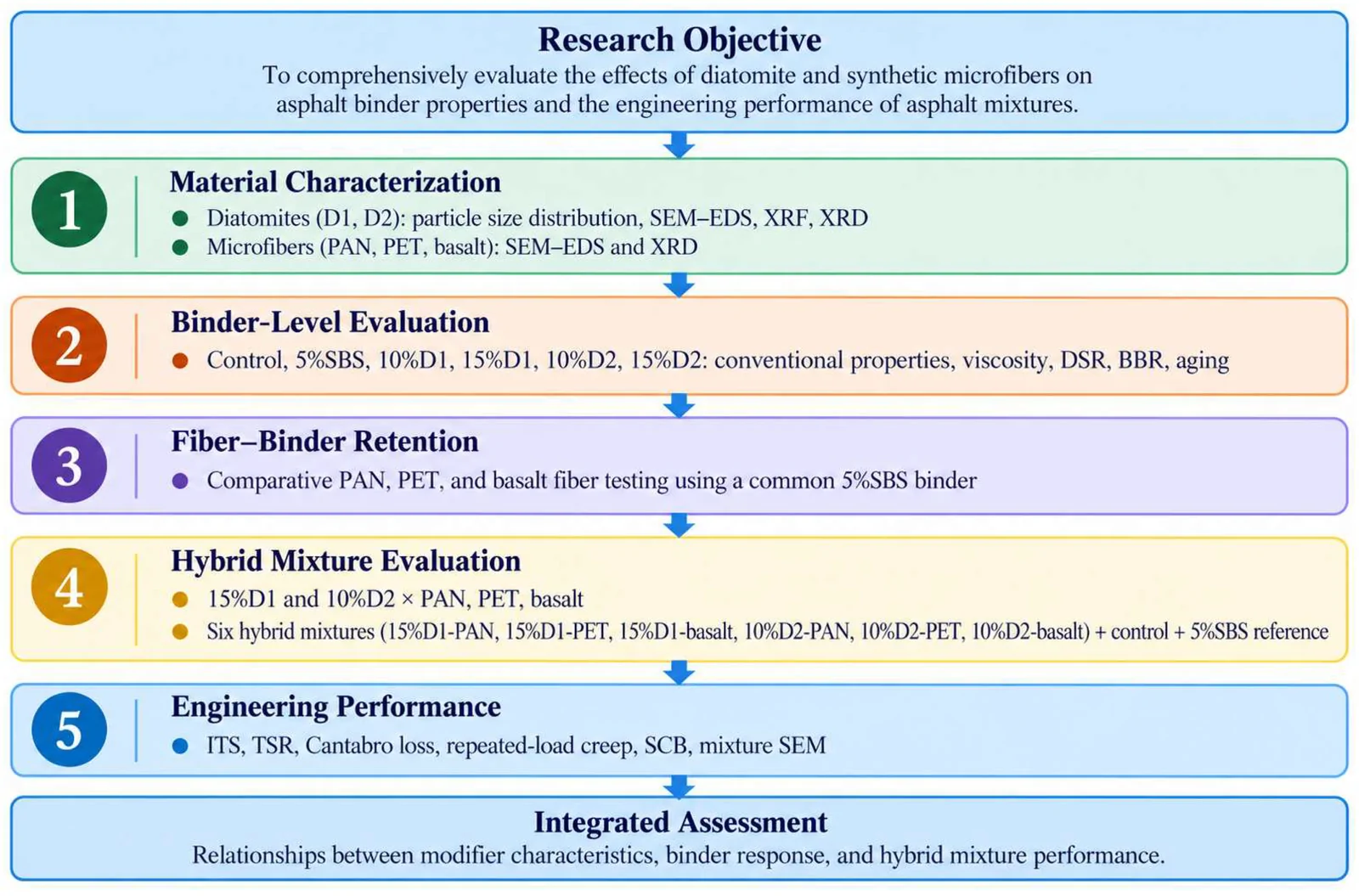
Schematic overview of the research objective and experimental workflow.

The experimental program was organized into five main stages, as illustrated in Figure 1. These stages included physicochemical characterization of the diatomites and microfibers, conventional and rheological evaluation of the asphalt binders and their aging behavior, comparative fiber–binder retention testing, preparation and characterization of the selected hybrid asphalt mixtures, and assessment of their engineering performance through mechanical testing and microstructural examination. The findings from these stages were considered together to evaluate the relationships between modifier characteristics, binder response, and mixture performance.

### 2.1. Materials

**Figure 2:**
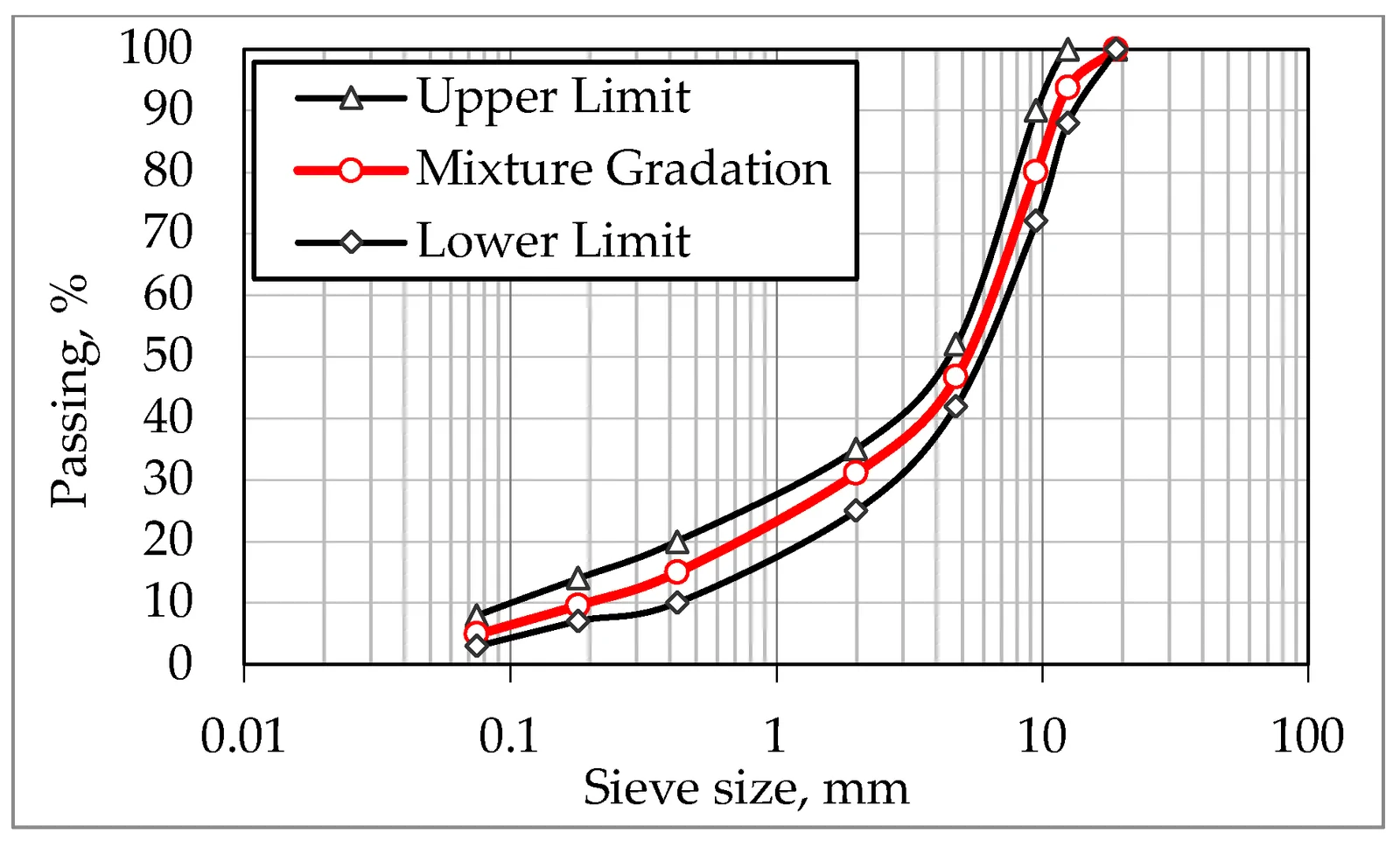
Aggregate gradation curve showing the combined aggregate gradation and the specified upper and lower gradation limits.

A 50/70 penetration-grade asphalt binder obtained from the Batman Refinery (Batman, Türkiye) was used as the base binder. Its basic physical properties and the corresponding specification limits are presented in Table 1. Basalt aggregate was used for the asphalt mixtures, and its principal physical and mechanical properties are summarized in Table 2. The aggregate gradation was selected in accordance with the Turkish Highway Technical Specifications [16] for dense-graded asphalt concrete. The adopted gradation and the corresponding specification limits are shown in Figure 2. The basalt mineral filler was obtained from Gülyurdu Quarry, Trabzon, Türkiye.

**Figure 3:**
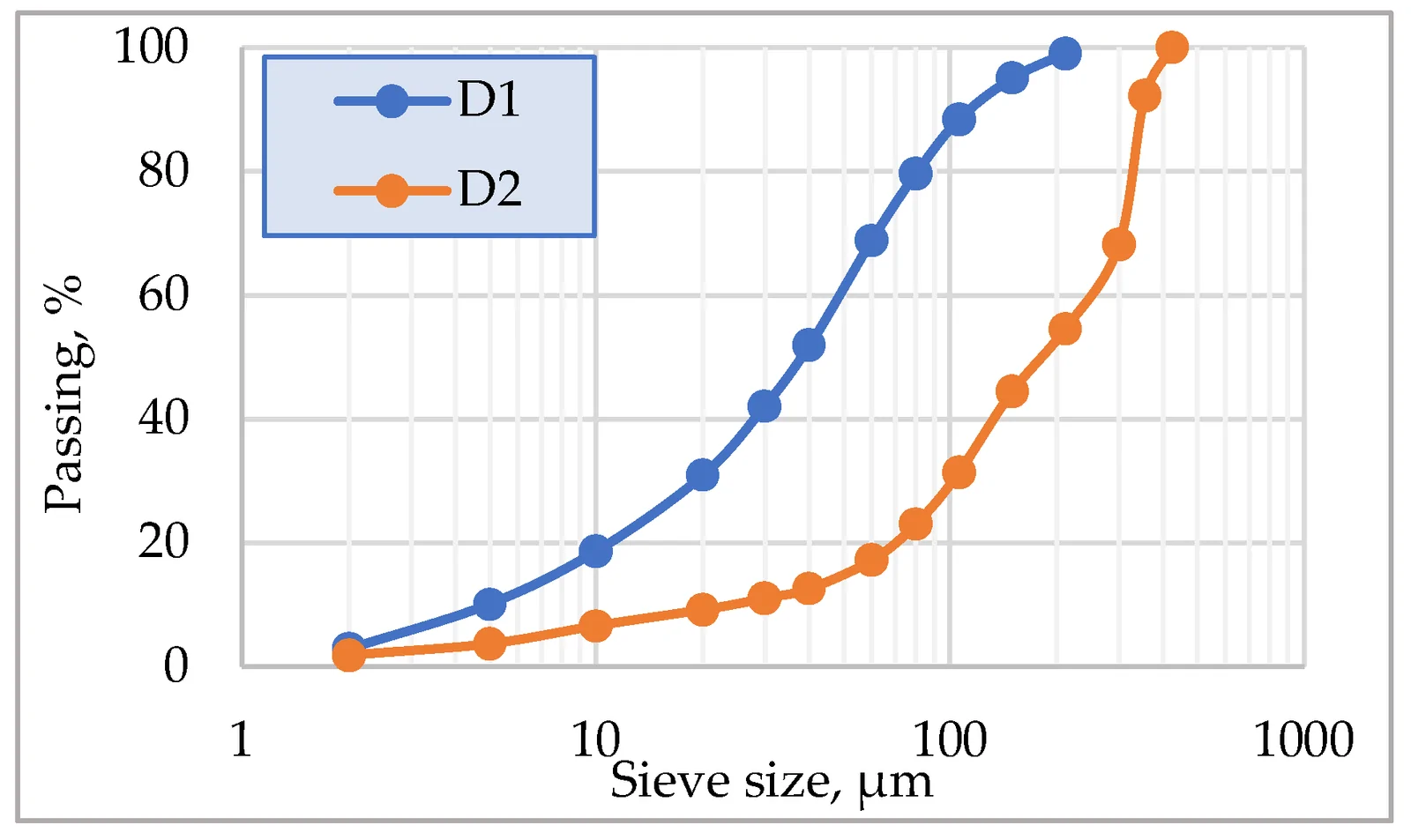
Particle size distribution curves of the two diatomites used in this study.

Two diatomite materials, designated D1 and D2, were supplied by Bentaş Bentonit A.Ş., Ordu, Türkiye and used as mineral modifiers. The maximum particle sizes of D1 and D2 were 212 and 425 µm, respectively. As shown in Figure 3, D1 exhibited a substantially finer particle-size distribution than D2. Diatomite is a naturally occurring siliceous material characterized by a porous structure and a relatively high binder-retention capacity, which makes it a potentially effective mineral modifier for asphalt binders and mixtures [7,8,29].

The investigated diatomite contents were selected based on the ranges reported in previous studies. Du et al. [30] reported an optimum diatomite content of approximately 13% and indicated that contents exceeding 20% could adversely affect performance. Zhang et al. [31] identified a suitable range of 10–13%, whereas Hou and Wu [32] reported improved rutting resistance at a diatomite content of 15%. Accordingly, both D1 and D2 were incorporated at 10% and 15% by mass of the base binder during the binder-characterization stage.

Polyacrylonitrile (PAN), polyethylene terephthalate (PET), and basalt microfibers supplied by Pioneer Scientific Industry Co., Ltd. (Nanjing, China) were used to produce the hybrid-modified asphalt mixtures. These fibers were selected because they represent reinforcement systems with distinctly different physical, mechanical, and compositional characteristics. PAN fibers have been investigated with respect to their interfacial adhesion characteristics with asphalt [13], whereas PET fibers provide a polymeric reinforcement system and have been investigated as reinforcement in asphalt concrete [14]. Basalt fibers, characterized by relatively high tensile strength and elastic modulus, have been reported to improve tensile strength, stripping resistance, dynamic stability, and fatigue performance [12]. The principal properties of the three microfiber types are summarized in Table 3.

The fiber contents were selected according to suitable or optimum ranges reported in previous studies. Basalt fibers have been shown to improve asphalt-mixture performance at contents of approximately 0.30% by aggregate mass [33,34]. Suitable contents of approximately 0.065% and 0.10% by aggregate mass have been reported for PAN and PET fibers, respectively [35,36]. Accordingly, PAN, PET, and basalt fibers were incorporated at 0.065%, 0.10%, and 0.30% by aggregate mass, respectively. These dosages were selected to evaluate each fiber within a previously reported suitable range rather than to establish a constant-concentration comparison among fiber types. Consequently, differences in mixture performance were interpreted as the combined effects of fiber type and dosage within each hybrid formulation, rather than as effects attributable solely to fiber type.

A linear styrene–butadiene–styrene (SBS) thermoplastic elastomer, Kraton D1192E, supplied by Kraton (Almere, The Netherlands), was used to prepare the polymer-modified reference binder. The polymer consists of rigid styrene blocks and flexible butadiene blocks, which provide thermoplastic and elastomeric characteristics, respectively. An SBS content of 5% by mass of the base binder was selected because this content is commonly used in conventional SBS-modified asphalt systems and is sufficient to promote polymer-network formation and measurable improvements in binder rheology and mixture performance [4]. The 5% SBS-modified system was therefore included as a conventional polymer-modified benchmark for evaluating the relative performance of the diatomite–microfiber hybrid systems. The principal technical properties of the SBS polymer are presented in Table 4.

### 2.2. Methods

#### 2.2.1. Asphalt Mixture Design and Preparation

The binder-level experimental program comprised the unmodified base binder, a 5% SBS-modified reference binder, and D1- and D2-modified binders containing 10% and 15% diatomite by mass of the base binder. For the mixture-level performance evaluation, 15%D1 and 10%D2 were selected as representative formulations within the diatomite dosage ranges supported by previous studies [30,31,32], allowing a focused assessment of their hybrid mixture performance within a manageable experimental matrix. Because diatomite source and content differed simultaneously between these formulations, their performance differences were interpreted as the combined responses of the complete systems rather than as the independent effects of diatomite type or dosage.

The diatomite-modified binders were prepared using the wet process. The base binder was heated to 180 °C, after which the required amount of diatomite was gradually added and dispersed using a high-shear mixer at 4500 rpm for 120 min. The 5% SBS-modified reference binder was prepared under the same temperature, mixing speed, and mixing duration conditions. The unmodified control binder was subjected to the same heating and high-shear mixing procedure (180 °C, 4500 rpm, and 120 min) to ensure an equivalent thermal and mechanical processing history across all binder formulations.

The asphalt mixtures were designed according to the Marshall method following ASTM D6926-20 [42] and ASTM D6927-22 [43]. A dense aggregate gradation conforming to the Turkish Highway Technical Specifications [16] was used, and the specimens were compacted by applying 75 blows to each face. For each binder system, the binder content corresponding to a target air-void content of 4% was adopted as the optimum binder content (OBC). For the fiber-reinforced mixtures, the OBC determined for the corresponding diatomite-modified mixture before fiber incorporation was retained without separate re-optimization for each fiber-containing formulation.

The fibers were incorporated using the dry process. The heated aggregates were first blended with the required amount of PAN, PET, or basalt fiber until a visually uniform distribution was obtained. The corresponding diatomite-modified binder was then added, and mixing continued until the aggregate and fibers were uniformly coated. The mixtures were subsequently conditioned and compacted using the Marshall procedure. The asphalt mixtures were prepared at a mixing temperature of 165 °C and compacted at 150 °C.

Six hybrid mixtures were produced by combining the 15%D1 and 10%D2 systems with PAN, PET, and basalt fibers. Together with the unmodified control and 5% SBS-modified reference, eight asphalt-mixture formulations were evaluated. Their compositions, fiber contents, OBC values, Marshall design parameters, and coding system are summarized in Table 5.

For the 15%D1 system, the VFA (76.18%) and VMA (16.34%) values slightly exceeded the specified upper limits of 75% and 16%, respectively. Nevertheless, this formulation was retained in the experimental program to enable comparative evaluation of the selected diatomite–microfiber hybrid systems. Comparable deviations in volumetric properties have been reported in previous asphalt-mixture studies. Le et al. [44] reported a VFA of 76.5% against an upper limit of 75%, while Villiers and Leon [45] reported VFA and VMA values of 76.28% and 18.9%, respectively, for a mixture containing recycled concrete aggregate. Similarly, Tan et al. [46] reported a VMA of 17.1% for a rubber–diatomite-modified asphalt mixture. These examples illustrate that modest deviations in volumetric parameters have also been documented in other experimental asphalt-mixture investigations, supporting the continued evaluation of the 15%D1 formulation for comparative research purposes.

#### 2.2.2. Microstructural, Crystallographic, and Chemical Analyses

The surface morphology and local elemental composition of the D1 and D2 diatomites, basalt filler, and PAN, PET, and basalt fibers were examined using scanning electron microscopy coupled with energy-dispersive X-ray spectroscopy (SEM–EDS). Before analysis, the materials were dried at 105 °C for 24 h and cooled to room temperature. They were then mounted on aluminum stubs using double-sided conductive carbon tape, and loose particles were removed using compressed air. To improve surface conductivity, the specimens were gold-coated for approximately 90 s using a Quorum Q150R ES Plus sputter coater (Quorum Technologies Ltd., Laughton, UK).

SEM imaging was performed using a Thermo Scientific Apreo 2S field-emission scanning electron microscope (Thermo Fisher Scientific Brno s.r.o., Brno, Czech Republic) at an accelerating voltage of 20 kV and at different magnification levels. EDS analyses were conducted on selected regions to determine localized semi-quantitative elemental compositions. For mixture-level SEM analysis, representative fragments were obtained from the control, 5% SBS-modified, and diatomite–microfiber hybrid Marshall specimens by cutting them with a precision saw. Sampling was performed from approximately the central regions of the compacted specimens. The extracted fragments were dried at 50 °C for 72 h and subsequently mounted and gold-coated using the procedure described above. One specimen was examined for each asphalt mixture formulation, with approximately two distinct fields of view assessed per specimen. The fields were purposively selected from regions where the fibers and surrounding asphalt matrix could be clearly distinguished, enabling qualitative observations of local features such as fiber embedment, clustering, matrix continuity, and interfacial morphology. Care was taken during specimen cutting to minimize preparation-induced damage and microcracking. The resulting SEM micrographs were interpreted as selected local observations rather than as evidence of uniform microstructural characteristics throughout the entire mixture.

The mineralogical and structural characteristics of the D1 and D2 diatomites, basalt filler, and PAN, PET, and basalt fibers were examined by X-ray diffraction (XRD). The analyses were conducted using a PANalytical X’Pert^3^ Powder diffractometer (PANalytical B.V., Almelo, The Netherlands) with Cu Kα radiation (λ = 1.5406 Å). The mineral samples were homogenized before being placed in suitable sample holders, while the fiber samples were mounted directly in the corresponding holders. The D1, D2, and basalt-filler samples were scanned over a 2θ range of 5–80° at 2°/min, whereas the PAN, PET, and basalt fibers were scanned over the same range at 1°/min. The detected diffraction features were evaluated by comparing their characteristic peak positions with the relevant entries in the ICDD Powder Diffraction File database.

The bulk chemical compositions of the D1 and D2 diatomites and basalt filler were determined by X-ray fluorescence (XRF) spectroscopy using a Rigaku ZSX Primus II spectrometer (Rigaku Corporation, Akishima, Tokyo, Japan) at the Middle East Technical University Central Laboratory (Ankara, Türkiye). Before analysis, the samples were dried at 105 °C for 24 h, cooled to room temperature, and placed in suitable holders without the addition of a binding agent. The basalt-filler and diatomite samples were analyzed using holders with diameters of 10 and 30 mm, respectively. Semi-quantitative measurements were performed over an elemental range from B to U, and the results were evaluated in both elemental and oxide forms.

#### 2.2.3. Characterization of Asphalt Binders

The conventional physical properties of the unmodified and modified asphalt binders were evaluated through density, penetration, and softening-point tests. Density was determined using a pycnometer with a capillary stopper in accordance with TS EN 15326+A1 [17], with two replicate measurements. Penetration was measured according to TS EN 1426 [20] at 25 ± 0.1 °C under a 100 g load applied for 5 s, and the mean of three measurements was reported. The softening point was determined using the ring-and-ball method according to TS EN 1427 [18], with an initial bath temperature of 5 ± 1 °C and a heating rate of 5 ± 0.5 °C/min. Two replicate measurements were conducted for each binder.

The temperature susceptibility of the binders was evaluated using the penetration index (PI), calculated from the penetration at 25 °C (Pen_25_) and the softening point (SP) according to Equation (1) [47]:

PI = (1952 − 500 × log_10_(Pen_25_) − 20 × SP)/(50 × log_10_(Pen_25_) − SP − 120) (1)

where (Pen_25_) is expressed in 0.1 mm and SP in °C. Higher PI values indicate lower temperature susceptibility.

The high-temperature flow behavior of the binders was evaluated using a rotational viscometer according to ASTM D4402 [48]. Measurements were performed at 135, 150, and 165 °C at a rotational speed of 20 rpm, and the mean of three stabilized readings was reported at each temperature. A viscosity limit of 3 Pa·s at 135 °C was adopted as the workability criterion.

Short-term aging was performed using the rolling thin-film oven test (RTFOT) according to TS EN 12607-1 [49]. Approximately 35 g of binder was placed in each bottle and aged at 163 °C for 85 min. Two replicate measurements were conducted for each binder. Long-term aging was subsequently applied to the RTFOT-aged binders using a pressure aging vessel (PAV) according to TS EN 14769 [50]. The binders were aged for 20 h under a pressure of 2070 kPa at the temperature corresponding to their performance grade.

The intermediate- and high-temperature rheological properties of the binders were evaluated using a Bohlin Gemini II dynamic shear rheometer (DSR; Malvern Instruments Ltd., Malvern, UK) in accordance with TS EN 14770 [51]. Measurements were conducted at an angular frequency of 10 rad/s. For the original and RTFOT-aged binders, a 25 mm parallel-plate geometry with a 1 mm gap was used at temperatures ranging from 58 to 82 °C in 6 °C increments, with strain levels of 12% and 10%, respectively. For the PAV-aged binders, an 8 mm parallel-plate geometry with a 2 mm gap and a strain level of 1% was employed, and measurements were performed at 25, 22, 19, 16, 13, and 10 °C. The high-temperature performance of the original and RTFOT-aged binders was evaluated using the G*/sin δ parameter, with minimum criterion values of 1.0 and 2.2 kPa, respectively, whereas the intermediate-temperature response of the PAV-aged binders was evaluated using G*·sin δ with a maximum criterion value of 5000 kPa, in accordance with AASHTO M 320-16 [52]. The corresponding critical temperatures were taken from the laboratory-reported DSR failure temperatures at the specified criterion limits. DSR measurements were performed using two independently prepared specimens for each binder formulation and aging condition. The critical temperatures reported in this study represent the mean values obtained from the two replicate measurements. The number of replicates was limited to two partly because of the restricted amount of binder available following the RTFOT and PAV aging procedures. Since the individual replicate results were not retained in the available laboratory report, measures of experimental variability, such as standard deviations, could not be reported.

The low-temperature rheological response of the RTFOT- and PAV-aged binders was evaluated using a bending beam rheometer (BBR) according to TS EN 14771 [53]. Beam specimens measuring 125 × 12.5 × 6.25 mm were subjected to a constant load of 980 mN for 240 s at −6, −12, and −18 °C, using two replicate beams for each binder at each test temperature. Creep stiffness (S) and the m-value were determined at 60 s and evaluated using the Superpave performance criteria of S ≤ 300 MPa and m ≥ 0.300 in accordance with AASHTO M 320-16.

#### 2.2.4. Fiber–Binder Retention Test

The relative binder-retention capacities of the PAN, PET, and basalt fibers were evaluated using an adapted basket-drainage procedure based on the methods reported by Kou et al. [54] and Shi et al. [55]. A common 5% SBS-modified binder was used for all fiber types to provide identical binder conditions and isolate the effect of fiber characteristics. Accordingly, the test was intended for comparative evaluation of the fibers rather than direct measurement of binder retention within the different diatomite–fiber mixtures.

Before testing, the fibers were dried at 105 ± 5 °C for 2 h to remove moisture. A fiber mass of 9 g (m_1_) was placed in a pre-weighed container and mixed with approximately 300 g of hot 5% SBS-modified binder until a visually uniform fiber–binder blend was obtained. The mass of the clean and dry metal drainage basket, which had openings of 0.315 mm, was recorded as (m_2_). The fiber–binder blend was then transferred to the basket, which was placed over a collection container and maintained in an oven at 165 °C for 12 h to allow excess binder to drain. After drainage, the total mass of the basket, fibers, and retained binder was recorded as (m_3_). The fiber–binder retention experiment was performed in two independent replicates for each fiber type (PAN, PET, and basalt).

The binder-retention ratio (BRR) was calculated using Equation (2):

(2) $$\mathrm{BRR}(\%)=\frac{m_{3}-m_{2}-m_{1}}{m_{1}}\times 100$$

where (m_1_) is the initial fiber mass (g), (m_2_) is the mass of the empty basket (g), and (m_3_) is the final mass of the basket, fibers, and retained binder (g). Because the measured binder may include both surface-adhered and internally absorbed fractions, the results were interpreted as comparative binder retention rather than intrinsic fiber absorption.

#### 2.2.5. Indirect Tensile Strength Test

The low-temperature tensile response of the asphalt mixtures was evaluated using the indirect tensile strength (ITS) test according to TS EN 12697-23 [56]. Cylindrical Marshall specimens with a nominal diameter of 101.6 mm were conditioned at 0 °C for 24 h to obtain a uniform test temperature. A diametral compressive load was then applied at a displacement rate of 50 mm/min until failure, and the peak load was recorded.

The ITS was calculated using Equation (3):

ITS = (2000 × P)/(π × D × t)(3)

where (ITS) is the indirect tensile strength (kPa), (P) is the peak load (N), (D) is the specimen diameter (mm), and (t) is the specimen thickness (mm). Three specimens were tested for each mixture, and the results were reported as the mean and standard deviation. Higher ITS values were interpreted as indicating a greater tensile load-carrying capacity under the applied test conditions [57,58].

#### 2.2.6. Moisture Susceptibility and Tensile Strength Ratio

The moisture susceptibility of the asphalt mixtures was evaluated according to AASHTO T 283-21 [59], using the tensile strength ratio (TSR). Six specimens were prepared for each mixture and divided equally into unconditioned and moisture-conditioned groups (*n* = 3 per group). The unconditioned specimens were maintained in a water bath at 25 °C for 2 h before testing.

The conditioned specimens were vacuum-saturated to achieve a degree of saturation of 70–80% and sealed in watertight bags for freezing. They were maintained at −18 °C for 16 h and subsequently immersed in a water bath at 60 °C for 24 h. Before testing, the conditioned specimens were equilibrated in a water bath at 25 °C for 2 h. Both conditioned and unconditioned specimens were then tested at 25 °C by applying a diametral compressive load at a displacement rate of 50 mm/min until failure.

The TSR was calculated from the mean ITS values of the conditioned and unconditioned groups using Equation (4):

(4) $$\mathrm{TSR}=\frac{{\mathrm{ITS}}_{\mathrm{conditioned}}}{{\mathrm{ITS}}_{\mathrm{unconditioned}}}\times 100$$

where ITS_conditioned_ and ITS_unconditioned_ represent the mean indirect tensile strengths of the conditioned and unconditioned specimens, respectively. Higher TSR values indicate greater retention of tensile strength after moisture conditioning. A minimum TSR value of 80% was adopted as the reference criterion in accordance with AASHTO M 323-13 [60], consistent with the criterion commonly applied in previous studies [61,62].

#### 2.2.7. Cantabro Particle-Loss Test

The resistance of the asphalt mixtures to particle loss was assessed using an adapted Cantabro test procedure based on TS EN 12697-17 [63]. Three Marshall specimens were prepared for each mixture and conditioned at 25 °C for at least 4 h prior to testing. Each specimen was weighed and individually placed in a Los Angeles abrasion drum without steel balls. The drum was operated for 300 revolutions at a rotational speed of 30 rpm. After testing, loose particles were removed, and the remaining specimen was weighed to determine the mass loss caused by the abrasion process.

The Cantabro loss (CL) was calculated using Equation (5):

(5) $$\mathrm{CL}\;(\%)=100\times \frac{\left(W_{1}-W_{2}\right)}{W_{1}}$$

where (CL) is the Cantabro mass loss (%), W_1_ is the specimen mass before testing (g), and W_2_ is the specimen mass after testing (g).

Three specimens were tested for each mixture, and the Cantabro loss values were reported as the mean and standard deviation. Lower Cantabro loss indicates greater resistance to particle loss under the applied test conditions. Because TS EN 12697-17 [63] is primarily intended for porous asphalt mixtures and no generally accepted Cantabro acceptance limit is available for dense-graded asphalt mixtures, the results were used only for comparative evaluation among the investigated mixtures rather than as a specification-based pass–fail criterion.

#### 2.2.8. Repeated-Load Creep Test

The permanent-deformation response of the asphalt mixtures was evaluated using the unconfined repeated-load creep test in accordance with Method A of TS EN 12697-25 [64]. Three Marshall specimens were prepared for each mixture and thermally conditioned at 40 °C for 8 h before testing. A seating stress of 3 kPa was initially applied for 2 min to ensure uniform contact between the specimen and loading system.

Following the seating stage, a repeated axial stress of 95 kPa was applied at a frequency of 0.5 Hz for 10,000 loading cycles. Cumulative permanent axial deformation was continuously recorded throughout the test. The permanent deformation measured at the end of 10,000 cycles was reported as the mean and standard deviation of three specimens, with lower deformation indicating greater resistance to repeated-load-induced permanent deformation under the applied test conditions [65,66,67].

#### 2.2.9. Semi-Circular Bending Test

The intermediate-temperature fracture response of the asphalt mixtures was evaluated using an adapted semi-circular bending (SCB) procedure based on AASHTO TP 124-18 [68]. Three cylindrical specimens with a nominal diameter of 100 mm and a thickness of 50 mm were prepared for each mixture. The use of 100 mm specimens was dictated by the available specimen preparation equipment at the time of testing, as the laboratory was equipped for preparing Marshall-compacted specimens but did not yet have a gyratory compactor capable of producing the nominal 150 mm specimens specified in AASHTO TP 124-18. A comparable modified SCB approach using 100 mm Marshall-compacted specimens and the same FI calculation framework was also employed in our previously published study [69]. Each specimen was cut into two equal semi-circular halves, producing six SCB specimens per mixture. A central notch with a depth of 15 ± 1 mm and a nominal width of 1.5 mm was introduced into each specimen using a diamond-blade cutting saw.

Before testing, the specimens were conditioned at 25 °C for 8 h. They were then positioned on two supports with a span of 80 mm and subjected to displacement-controlled monotonic loading at a rate of 50.8 mm/min. Load and load-line displacement (P–LLD) data were continuously recorded until failure. The applied loading rate of 50.8 mm/min corresponds to 2 in./min and is approximately equivalent to the nominal rate of 50 mm/min specified in AASHTO TP 124-18.

The fracture work (W_f_) was determined by numerical integration of the area under the P–LLD curve and was expressed in N·mm. The ligament area (A_lig_) was calculated using Equation (6):

A_lig_ = (r − a) × t (6)

where (r) is the specimen radius (mm), (a) is the notch length (mm), and (t) is the specimen thickness (mm). Fracture energy (G_f_) was calculated using Equation (7):

(7) $$G_{f}=\frac{W_{f}}{A_{\mathrm{lig}}}\times 10^{3}$$

where (W_f_) is the fracture work expressed in N·mm, (A_lig_) is the ligament area expressed in mm^2^, and (G_f_) is the fracture energy expressed in J/m^2^.

The flexibility index (FI) was determined from the fracture energy and the absolute value of the post-peak slope at the inflection point of the P–LLD curve according to Equation (8):

(8) $$\mathrm{FI}=\frac{G_{f}}{\left|m\right|}\times 0.01$$

where (m) is the post-peak slope determined according to AASHTO TP 124-18 [68], and 0.01 is the scaling coefficient. A recent independent investigation also applied the same FI formulation and the 0.01 scaling coefficient to SCB specimens with a diameter of 100 mm [70]. The coefficient of 0.01 was retained as part of the published FI formulation and was not used as a geometry-correction factor to establish equivalence between the 100 mm and 150 mm specimen configurations. Higher (G_f_) and FI values were interpreted as indicating greater energy-absorption capacity and greater resistance to post-peak crack propagation under the applied test conditions. The results were reported as the mean and standard deviation of six SCB specimens per mixture. Because the SCB specimens used in this study had a diameter of 100 mm and a notch depth of 15 ± 1 mm, the resulting G_f_ and FI values were used for internal comparisons among the investigated mixtures and should not be considered directly interchangeable with values obtained using the standardized AASHTO TP 124-18 specimen configuration [71,72]. Accordingly, no acceptance thresholds established for the standard 150 mm SCB configuration were applied to the FI values obtained from the adapted 100 mm specimens. The observed differences in G_f_ and FI were interpreted descriptively, as statistical significance testing was not performed.

## 3. Results and Discussion

### 3.1. Physicochemical and Microstructural Characterization of Modifiers

#### 3.1.1. SEM–EDS Analysis

**Figure 4:**
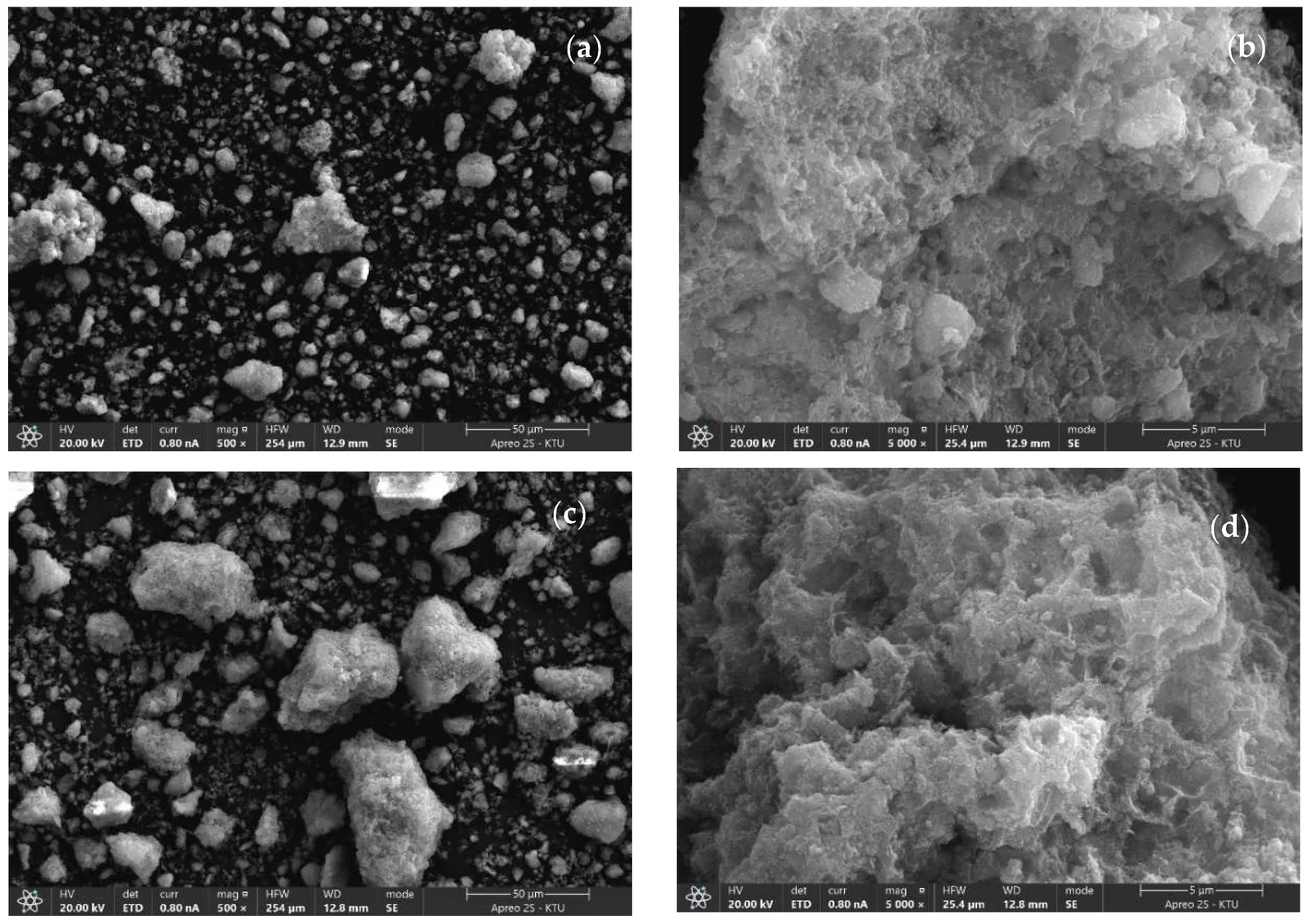
SEM micrographs of the diatomites: (**a**,**b**) D1 and (**c**,**d**) D2 at 500× and 5000× magnifications, respectively.

Representative SEM micrographs of the D1 and D2 diatomites are presented in Figure 4, while additional images obtained at different magnifications are provided in Figures S1 and S2. Both diatomites consisted of irregularly shaped particles with rough, porous surfaces and localized agglomerations. D1 generally exhibited finer and relatively more uniform particles, consistent with its particle-size distribution, whereas D2 showed a broader range of particle sizes and more pronounced surface irregularities. The porous morphologies observed in both materials may facilitate physical interaction with asphalt binder and retention of some binder fractions [73]. However, specific surface area, pore-size distribution, and binder–mineral surface interactions were not directly measured using BET analysis, porosimetry, or surface energy measurements. Therefore, the observed surface morphology cannot establish binder absorption capacity, interfacial adhesion, or the mechanisms responsible for the measured rheological and engineering responses.

**Figure 5:**
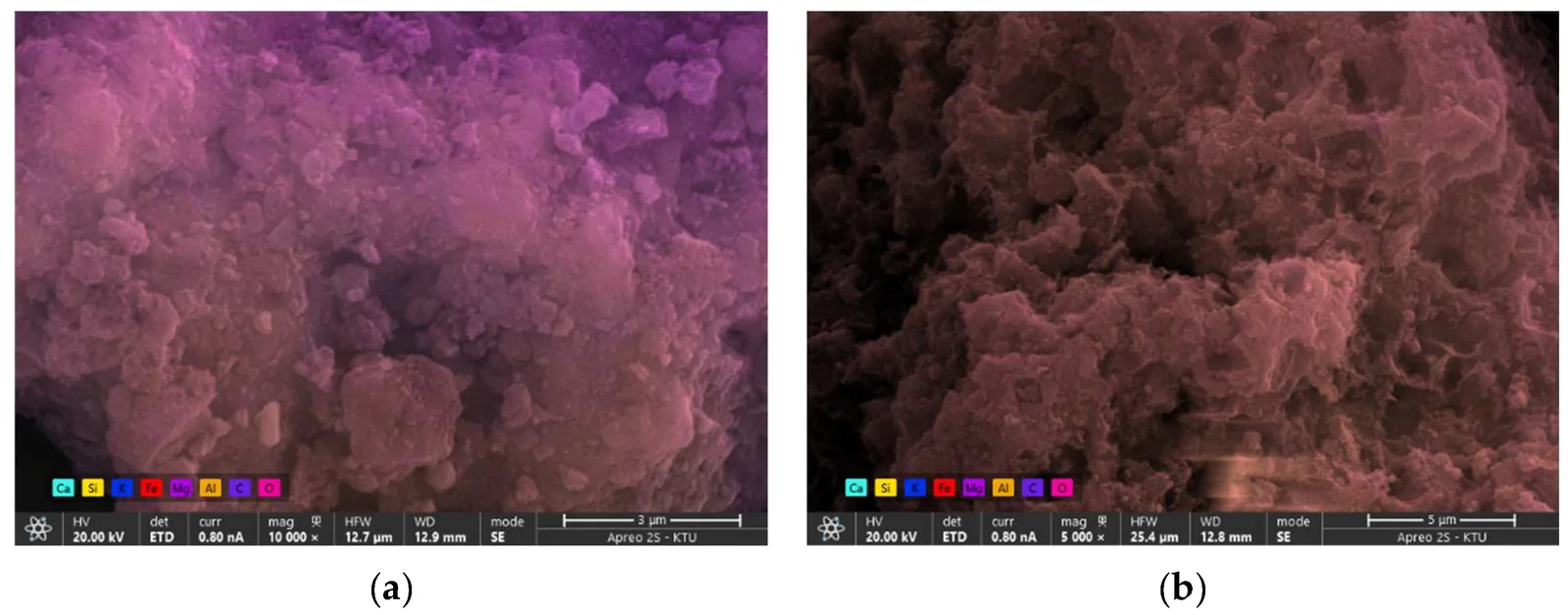
SEM micrographs showing the regions examined by EDS for the (**a**) D1 and (**b**) D2 diatomite samples.

EDS analyses were performed over the regions shown in Figure 5. The corresponding semi-quantitative elemental compositions are summarized in Table 6, and the EDS spectra are provided in Figure S3. Both diatomites exhibited Si- and O-rich local compositions. Within the analyzed regions, D2 contained 22.1 at.% and 32.9 wt.% Si, whereas D1 contained 10.4 at.% and 18.0 wt.% Si. Smaller amounts of Al, K, Ca, Fe, and Mg were also detected. These elements are compatible with the mineral constituents reported for natural diatomites, although the present EDS measurements alone do not establish the specific mineral phases responsible for their occurrence [74].

The measured carbon contents differed considerably between D1 and D2. However, because the specimens were mounted on conductive carbon tape, the carbon signal may have been affected by differences in particle coverage over the substrate. Carbon was therefore not used as evidence of an intrinsic compositional difference between the diatomites. Moreover, EDS provides localized surface information and should not be regarded as representative of bulk chemical composition. Accordingly, the bulk compositional comparison of D1 and D2 was based primarily on the XRF results presented in Section 3.1.2.

The elemental maps support the Si- and O-rich character of both diatomites and indicate a comparatively more uniform distribution of these elements within the examined D2 region. Nevertheless, EDS does not directly determine surface energy, adhesion, binder retention, or mechanical performance. The observed elemental differences were therefore interpreted together with the XRF, XRD, binder, and mixture-level results rather than as independent evidence of performance mechanisms.

The basalt filler exhibited irregular, angular, and fractured particles with rough surfaces and a heterogeneous particle-size distribution. Its EDS spectrum was dominated by Si, Mg, Al, Fe, and Ca, consistent with an aluminosilicate-based mineral composition. These characteristics may contribute to physical contact and internal friction within the asphalt mastic; however, adhesion and moisture resistance cannot be determined directly from SEM–EDS. Because basalt filler was common to all mixtures and was not an experimental variable, its detailed SEM images, EDS map, and elemental composition are provided in Figures S4 and S5 and Table S1, respectively.

**Figure 6:**
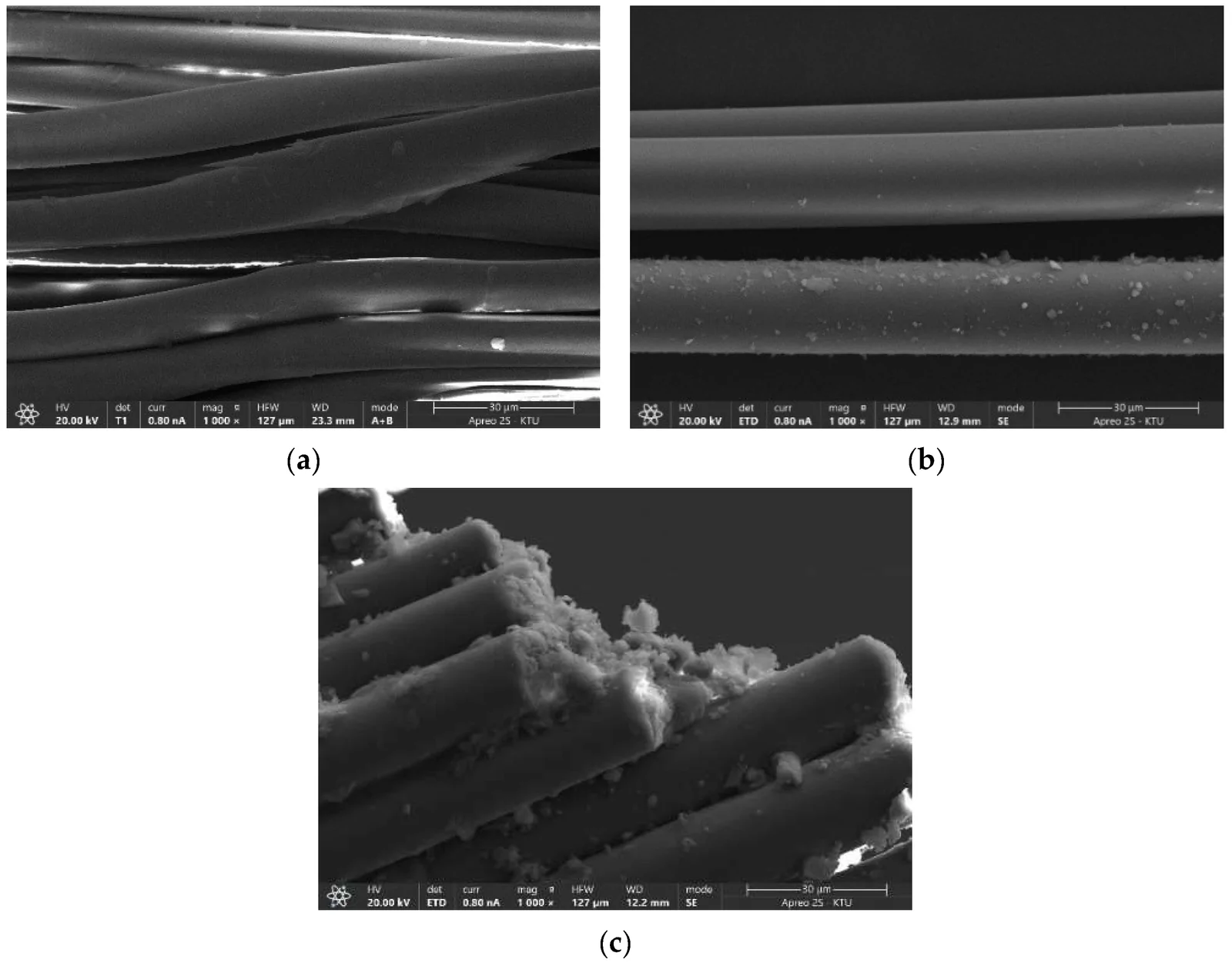
SEM micrographs of the microfibers at 1000× magnification: (**a**) PAN, (**b**) PET, and (**c**) basalt fiber.

Representative SEM micrographs of the PAN, PET, and basalt fibers are presented in Figure 6, with additional magnifications provided in Figures S6–S8. The PAN fibers exhibited irregular surfaces and locally fractured features, whereas the PET fibers showed comparatively smoother and more uniform surfaces. Basalt fibers appeared as cylindrical filaments arranged in approximately parallel bundles, with a measured diameter of approximately 19.57 µm, which was within the manufacturer-reported range. Their surfaces were relatively smooth, although local irregularities and damaged fiber ends were observed. These observations establish clear differences in the surface morphology of the three fiber types. However, the isolated-fiber SEM images do not provide direct evidence of fiber dispersion, crack bridging, pull-out, or interfacial load transfer within asphalt mixtures.

**Figure 7:**
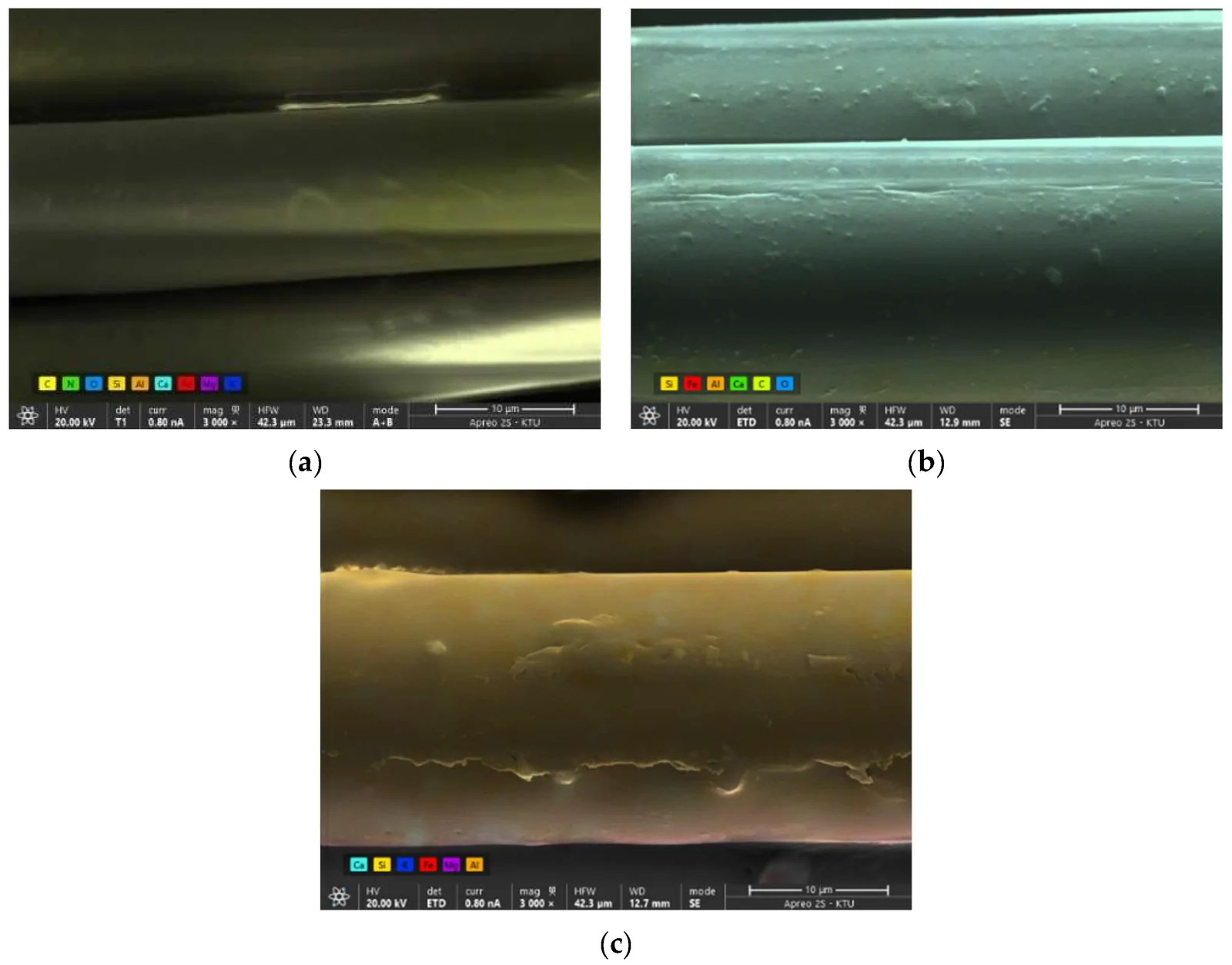
Combined EDS elemental maps of the microfibers: (**a**) PAN, (**b**) PET, and (**c**) basalt fiber.

The combined EDS maps and the main semi-quantitative elemental compositions of the fibers are presented in Figure 7 and Table 7, respectively, while the corresponding EDS spectra and detailed elemental compositions, including atomic and weight percentages, are provided in Figure S9 and Table S2, respectively. PAN was composed predominantly of C and N, consistent with the nitrogen-containing structure of polyacrylonitrile. PET was dominated by C and O, reflecting its polyester-based composition. In contrast, basalt fiber exhibited an inorganic composition dominated by Si and Al, together with Ca, Fe, Mg, and K. The EDS results therefore clearly distinguish the polymeric PAN and PET fibers from the aluminosilicate-based basalt fiber.

Overall, the SEM–EDS analyses revealed distinct morphological and elemental characteristics among the investigated diatomites and microfibers. D1 exhibited relatively finer and more uniform particles than D2, while the PAN, PET, and basalt fibers differed in surface morphology and elemental composition. These findings establish the principal material-level differences relevant to interpreting the subsequent binder-retention and mixture-performance results. However, SEM–EDS alone does not quantify interfacial adhesion, fiber dispersion within asphalt mixtures, or mechanical reinforcement.

#### 3.1.2. XRF Analysis

The XRF spectra of D1 and D2 are presented in Figure S10, while their complete elemental and oxide compositions are provided in Tables S3 and S4, respectively. The major oxide components are summarized in Table 8. Both diatomites were characterized by silica-rich bulk compositions. D1 and D2 contained 72.1 and 69.2 wt.% SiO_2_, respectively, followed by similar amounts of Al_2_O_3_ (9.02 and 9.47 wt.%), Fe_2_O_3_ (3.31 and 3.15 wt.%), MgO (3.01 and 3.02 wt.%), and CaO (2.30 and 2.50 wt.%). Their K_2_O contents were also comparable, at 1.22 and 1.33 wt.%, respectively. These results indicate that D1 and D2 had broadly similar bulk chemical compositions, although D1 contained a moderately higher proportion of SiO_2_.

The predominance of SiO_2_ is consistent with the siliceous character of natural diatomite and supports the XRD results presented in Section 3.1.3 [75]. The comparable Al_2_O_3_, Fe_2_O_3_, MgO, and CaO contents further indicate that the principal difference between the two materials was not a major change in bulk oxide composition. Although Ca- and Mg-containing components may influence mineral–binder interactions, XRF does not directly quantify surface chemistry, adhesion, binder retention, or aging behavior. These mechanisms should therefore be interpreted together with the morphological, mineralogical, rheological, and mixture-level results rather than inferred from XRF alone. The basalt filler, which was common to all mixtures, exhibited a silicate-based composition dominated by SiO_2_, Al_2_O_3_, Fe_2_O_3_, MgO, and CaO. Its XRF spectrum and complete elemental and oxide compositions are provided in Figure S11 and Tables S5 and S6, respectively. Because basalt filler was not an experimental variable, its chemical composition was used only as supporting material-characterization information.

The XRF results demonstrate that D1 and D2 were chemically similar, silica-rich mineral modifiers, with D1 exhibiting a slightly higher SiO_2_ content. Accordingly, the differences observed in binder and mixture performance cannot be attributed to bulk chemical composition alone and should be evaluated together with differences in particle-size distribution, morphology, mineralogical structure, modifier content, and fiber type.

#### 3.1.3. XRD Analysis

**Figure 8:**
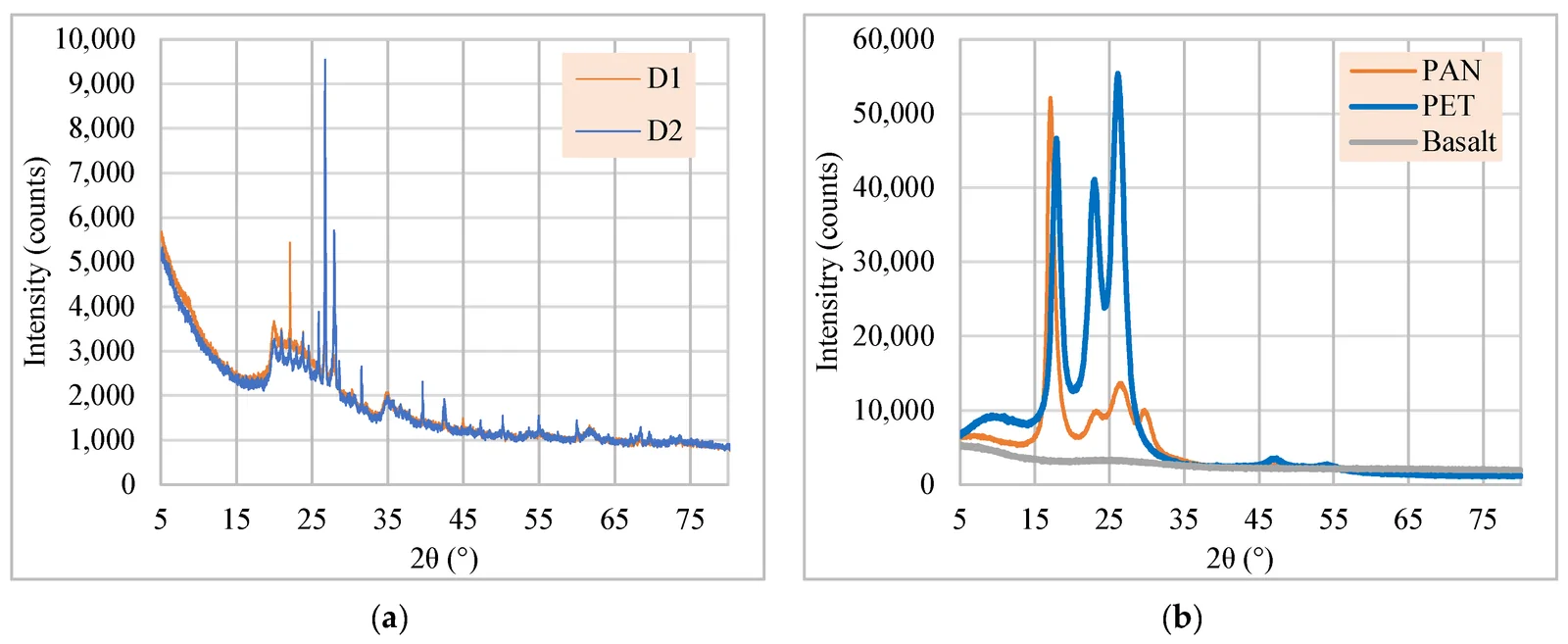
XRD patterns of the investigated modifiers: (**a**) D1 and D2 diatomites and (**b**) PAN, PET, and basalt fibers.

The XRD patterns of the two diatomites are presented in Figure 8a. Both D1 and D2 exhibited a broad diffuse halo in the 20–30° 2θ range together with distinct crystalline reflections. This pattern is consistent with the coexistence of amorphous silica, commonly associated with opal-A, and crystalline mineral phases in natural diatomite [76]. The prominent reflection at approximately 26.6° 2θ was assigned to quartz, while the weaker reflections were tentatively attributed to feldspar and other minor mineral phases. D1 and D2 displayed broadly similar siliceous mineralogical characteristics. The diffuse halo appeared more pronounced in D1, whereas the quartz reflection near 26.6° 2θ was more prominent in D2. These observations indicate qualitative differences in the mineralogical structure of the two diatomites. The XRF results confirmed the silica-rich bulk composition of both materials but did not distinguish between amorphous and crystalline silica. Because the XRD patterns were not intensity-normalized and no Rietveld refinement, internal-standard method, or quantitative amorphous-phase analysis was performed, the relative amorphous and crystalline contents of D1 and D2 could not be quantified. Therefore, the differences observed in the diffraction patterns were interpreted qualitatively. The basalt filler exhibited reflections tentatively associated with plagioclase- and pyroxene-group minerals, together with a broad background contribution indicative of poorly crystalline or glassy material. The complete XRD pattern of the basalt filler is provided in Figure S12 in the Supplementary Materials.

The XRD patterns of the PAN, PET, and basalt fibers are compared in Figure 8b. The basalt fiber exhibited a low-intensity, predominantly diffuse diffraction pattern without pronounced sharp reflections, consistent with the largely amorphous structure commonly reported for basalt fibers [77]. The PAN fiber displayed a pronounced diffraction peak near 17° 2θ and a weaker, broader feature around 26–27° 2θ. The peak near 17° is consistent with the characteristic diffraction response reported for PAN precursor fibers [78]. The higher-angle contribution may include amorphous scattering and cannot be assigned exclusively to a crystalline reflection without further analysis. In contrast, the PET fiber exhibited distinct diffraction peaks at approximately 18°, 23°, and 26° 2θ, consistent with the presence of ordered crystalline domains in semicrystalline PET [79].

Because the patterns were not normalized and no quantitative crystallinity analysis was conducted, absolute peak intensities and crystallinity levels were not directly compared among the fiber types. The XRD findings were therefore used to identify their principal structural characteristics rather than to establish direct causal relationships with the mechanical performance of the asphalt mixtures. The possible effects of these structural differences were evaluated together with the morphological, retention, and mechanical-test results.

### 3.2. Fiber–Binder Retention and Interaction Potential

**Figure 9:**
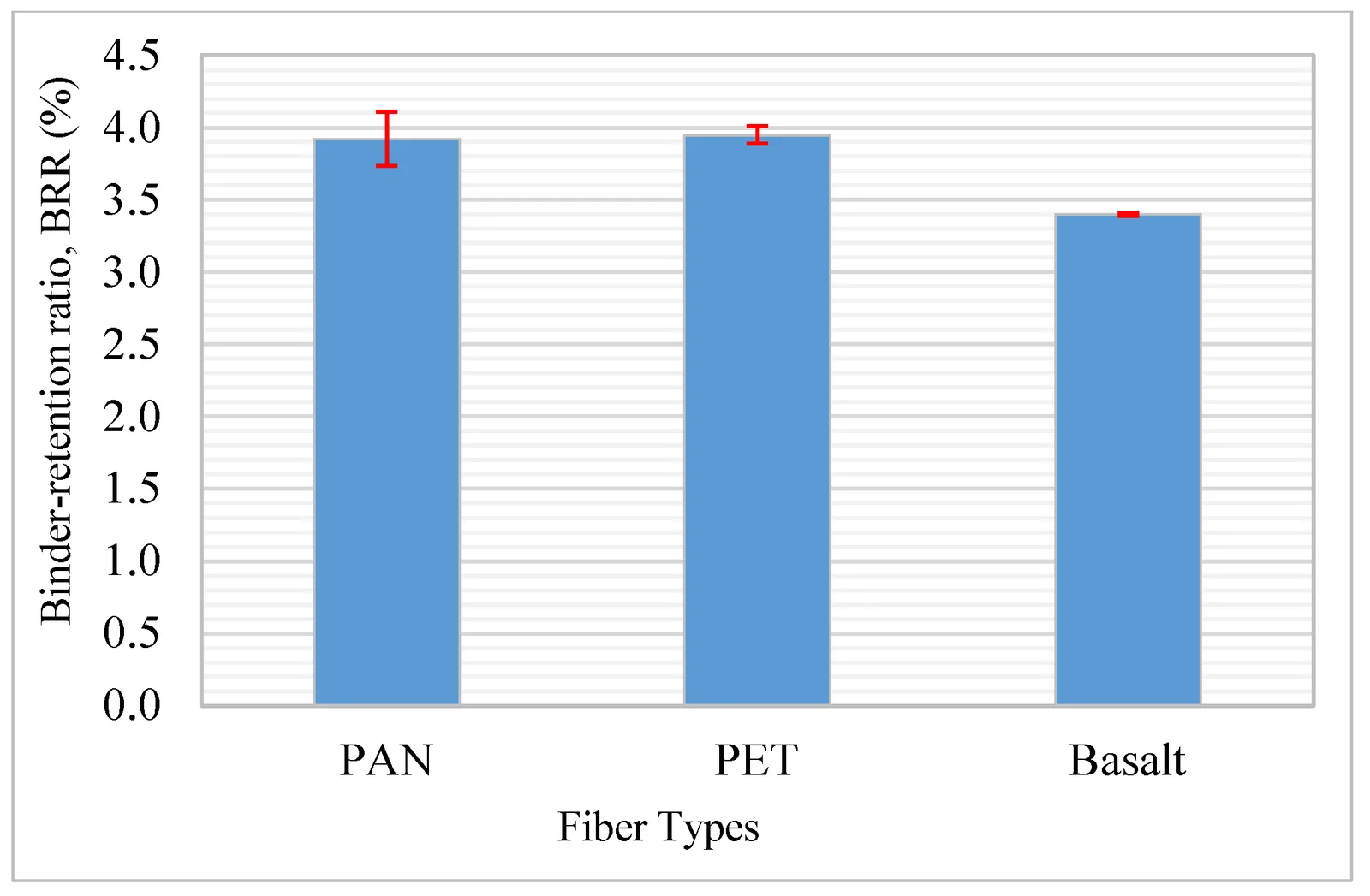
Binder-retention ratios of the PAN, PET, and basalt fibers.

The binder-retention ratios (BRRs) of the PAN, PET, and basalt fibers are presented in Figure 9. Under identical testing conditions using the 5% SBS-modified binder, the measured BRR values were 3.92%, 3.95%, and 3.40% for the PAN, PET, and basalt fibers, respectively. PET and PAN exhibited nearly identical retention ratios, whereas the basalt fiber produced a comparatively lower value. These results demonstrate differences in the relative amount of binder retained by the investigated fiber types but do not, in the absence of statistical testing, establish statistically significant differences.

The relatively high and similar BRR values of the PET and PAN fibers may be associated with their surface morphologies. The irregularities observed in the SEM micrographs could provide additional sites for binder attachment and mechanical interlocking. In contrast, the smoother and more compact appearance of the basalt-fiber surface may have contributed to its lower binder-retention ratio. However, because specific surface area, porosity, surface energy, and chemical adhesion were not directly measured, these explanations should be regarded as potential mechanisms rather than confirmed causal relationships. Moreover, the measured BRR includes both surface-adhered and internally absorbed binder and should not be interpreted as an intrinsic absorption capacity. Binder retention can affect the distribution and effective availability of the binder within fiber-reinforced asphalt mixtures. Greater retention may contribute to binder stabilization and reduce binder mobility, whereas excessive retention could decrease the amount of effective binder available for coating the aggregate particles and influence mixture workability [54,55,80,81]. Nevertheless, the results obtained using the common 5% SBS-modified binder provide a comparative characterization of the fibers and cannot independently predict their effects within the different diatomite-modified mixtures. Fiber geometry, dosage, dispersion, mechanical properties, and interactions with the surrounding mastic must also be considered when interpreting mixture performance.

A limitation of the mixture design is that the optimum binder content was determined for the corresponding diatomite-modified mixtures before fiber incorporation and was not independently re-optimized for each fiber-reinforced system. Although the fibers were incorporated at relatively low dosages, their different binder-retention characteristics may have altered the amount of effective binder available in the mixtures. Therefore, differences among the PAN-, PET-, and basalt-fiber mixtures may reflect both fiber-reinforcement mechanisms and variations in effective binder availability. Future studies should independently optimize the binder content of each fiber-modified system to quantify the influence of fiber-specific binder demand.

### 3.3. Microstructure of Diatomite–Fiber Systems

**Figure 10:**
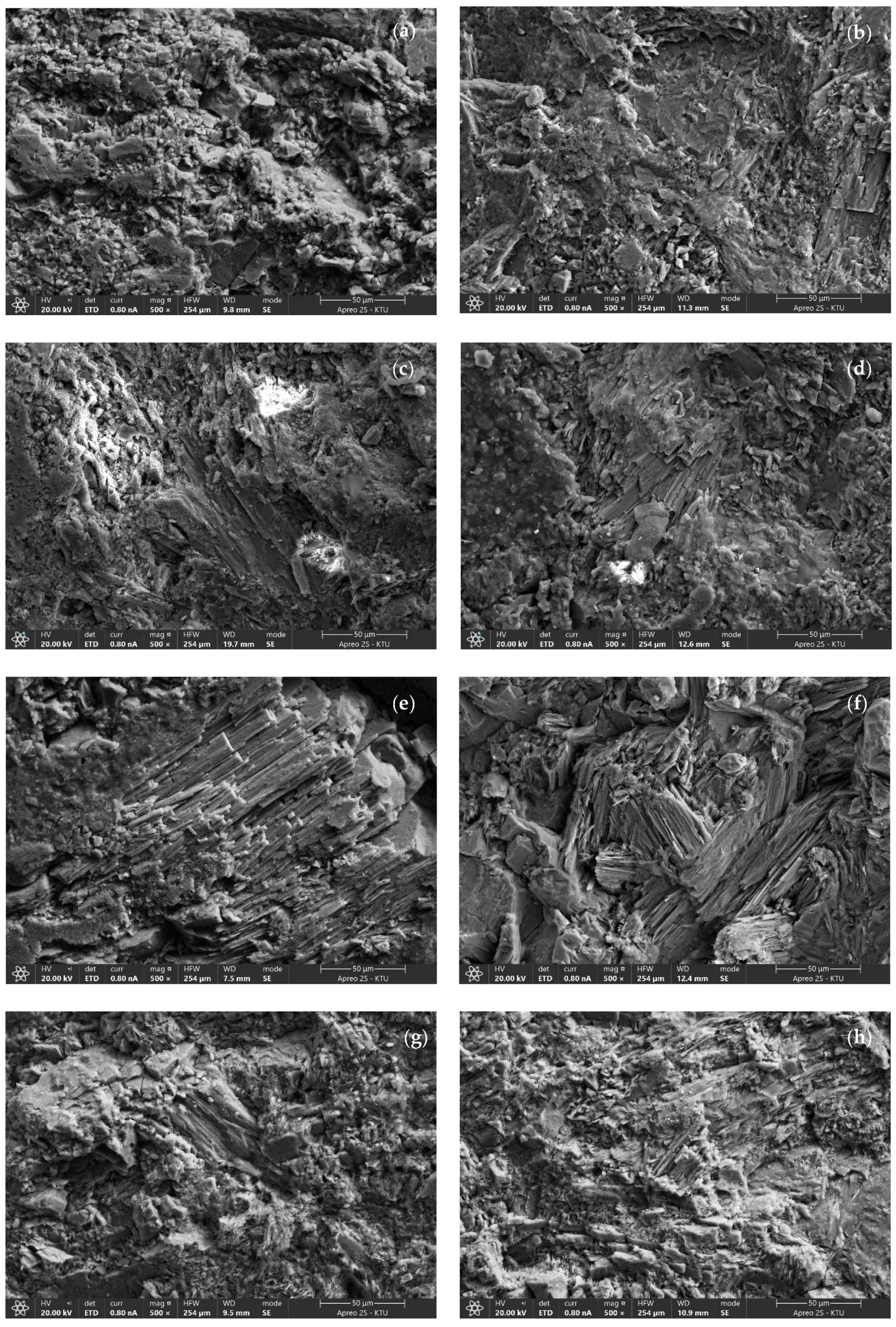
Representative SEM micrographs obtained at 500× magnification: (**a**) unmodified control; (**b**) 5% SBS-modified reference; (**c**–**e**) 15%D1 mixtures containing PAN, PET, and basalt fibers, respectively; and (**f**–**h**) 10%D2 mixtures containing PAN, PET, and basalt fibers, respectively.

Representative SEM micrographs of the unmodified control, 5% SBS-modified reference, and the six hybrid mixtures produced using 15%D1 and 10%D2 are compared in Figure 10. These two diatomite formulations were selected to represent contrasting D1- and D2-based systems while maintaining a manageable mixture-level experimental matrix. All images presented in Figure 10 were obtained at the same magnification, while additional SEM images at different magnifications are provided in Figures S13–S20.

The examined region of the unmodified control exhibited a relatively heterogeneous morphology, with localized discontinuities and microvoids at the aggregate–binder interface (Figure 10a). In contrast, the 5% SBS-modified reference displayed a comparatively more continuous and compact matrix in the examined field (Figure 10b). However, the SEM images alone neither demonstrate the formation of an SBS polymer network nor quantify its contribution to the viscoelastic response of the mixture.

The 15%D1-PAN and 15%D1-PET mixtures showed fiber-like features that appeared relatively well embedded within the surrounding matrix, with generally continuous local contact between the fibers and adjacent material (Figure 10c,d). Such embedment may facilitate fiber–matrix interaction when adequate interfacial bonding is present, although the SEM observations alone do not quantify interfacial adhesion or stress transfer [82,83]. The examined region of the 15%D1-BF mixture contained more pronounced local fiber clustering, fractured fiber ends, and localized separation features (Figure 10e). These characteristics may reflect local differences in fiber dispersion and interfacial morphology, although some discontinuities could also have resulted from specimen cutting or fracture-surface preparation. The corresponding 10%D2-PAN, 10%D2-PET, and 10%D2-BF mixtures also exhibited heterogeneous fiber–matrix morphologies (Figure 10f–h). The PAN-containing region showed relatively continuous embedded fiber features, whereas the PET fibers appeared comparatively smooth and locally oriented. The basalt fibers retained their elongated morphology but exhibited localized clustering and fractured ends. These observations indicate that fiber type may influence the local configuration and dispersion of the reinforcing phase within the hybrid mixtures. They also emphasize the importance of fiber geometry, dispersion, and mixing conditions in the development of fiber-reinforced asphalt microstructures [10,84]. Differences in local matrix continuity, fiber embedment, and clustering were observed between the examined 15%D1 and 10%D2 fields. However, diatomite type and dosage differed simultaneously between these systems; therefore, the observed features cannot be attributed independently to either factor. In addition, the SEM images represent selected local fields and do not provide quantitative measurements of overall fiber dispersion, interfacial bond strength, or load-transfer efficiency. Accordingly, the SEM observations, obtained from purposively selected local fields of view, were interpreted as qualitative supporting evidence alongside the binder-retention, rheological, and mechanical-performance results rather than as direct proof of the mechanisms governing mixture performance.

### 3.4. Physical and Rheological Characteristics of the Binders

The conventional physical properties of the unmodified and modified binders are summarized in Table 9. Diatomite modification increased the specific gravity of the binder, with values ranging from 1.082 to 1.119. The D1-modified binders generally exhibited higher specific gravity values than the corresponding D2 systems, whereas the 5% SBS-modified binder had a specific gravity of 1.014. The increase in specific gravity is consistent with the incorporation of mineral particles into the binder. The differences between the D1- and D2-modified binders may also reflect variations in the physical characteristics of the two diatomites; however, the present measurements do not isolate the individual contributions of particle density, morphology, and other material properties. The penetration and softening-point results also indicated an overall increase in binder consistency following modification. Compared with the unmodified binder, the modified binders generally exhibited lower penetration and higher softening-point values, with the greatest change observed for 5%SBS. The penetration index increased from −0.807 for the unmodified binder to values ranging from −0.364 to 0.469 for the diatomite-modified binders and reached 1.782 for 5%SBS. However, the response of the D1- and D2-modified binders was not proportional to diatomite content, indicating that binder consistency and temperature susceptibility depended on the combined effects of diatomite type and dosage.

**Figure 11:**
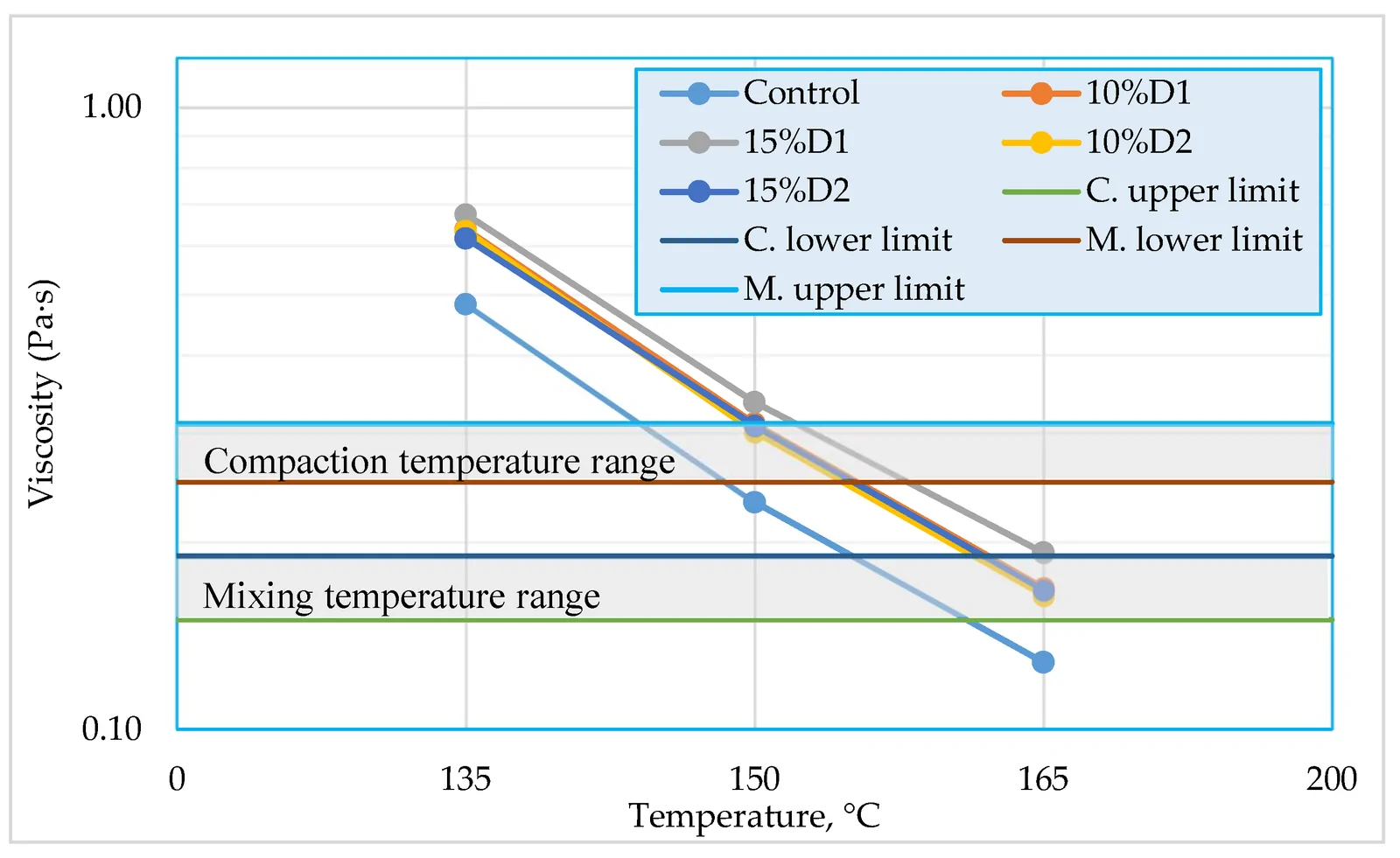
Rotational viscosities of the unmodified and diatomite-modified binders at different temperatures.

The rotational-viscosity results are presented in Figure 11. At 135 °C, the viscosity of the unmodified binder was 0.4825 Pa·s, whereas the diatomite-modified binders exhibited values ranging from 0.6158 to 0.6725 Pa·s. The highest viscosity was obtained for 15%D1. The viscosity of all binders decreased with increasing temperature, as expected. The increase in viscosity may be associated with the presence of diatomite particles and their physical interactions with the asphalt binder, as suggested in previous studies of diatomite-modified asphalt [73]. However, the present viscosity measurements do not independently establish the underlying interaction mechanism. Increasing the D1 content from 10% to 15% increased viscosity, whereas the same dosage increase produced a slight reduction in the D2 system. This contrasting response further indicates that the influence of diatomite was dependent on both its source and dosage. Nevertheless, all evaluated binders remained below the 3 Pa·s viscosity limit at 135 °C, indicating that the increase associated with diatomite addition did not reach a critical level for workability under the investigated conditions.

The DSR and aging results are summarized in Table 10. Based on the G*/sin δ > 1.0 kPa criterion, the critical temperature of the unaged binder increased from 67.2 °C for the unmodified binder to 70.5, 69.6, 69.1, and 67.8 °C for 10%D1, 15%D1, 10%D2, and 15%D2, respectively. The highest value among the diatomite-modified binders was obtained for 10%D1, whereas 5%SBS produced the highest overall critical temperature of 82.5 °C. These results indicate that diatomite modification can improve high-temperature, rutting-related rheological performance, although increasing the diatomite content did not produce a proportional improvement [29,74].

After RTFOT aging, the critical temperature determined using the (G*/sin δ >2.2) kPa criterion was 66.3 °C for the unmodified binder and ranged from 66.1 to 69.3 °C for the diatomite-modified binders. The highest diatomite-based value was obtained for 15%D1, while 5%SBS retained the highest overall value at 79.0 °C. The reported mass changes remained low for all binders, ranging from 0.05% to 0.30%. Penetration retention and softening-point changes varied with modifier type and dosage. In particular, 10%D1 exhibited the highest penetration retention among the investigated binders, at 87%, whereas the comparatively greater softening-point increases in some diatomite-modified binders indicated that their short-term aging response was sensitive to diatomite characteristics and content [8]. Following PAV aging, the critical temperatures determined using the (G*·sin δ < 5000) kPa criterion were 28.2, 30.9, 29.6, 28.8, 27.1, and 24.2 °C for the unmodified, 10%D1, 15%D1, 10%D2, 15%D2, and 5%SBS binders, respectively. The lower critical temperature of 5%SBS indicated a more favorable intermediate-temperature rheological response after long-term aging. In the BBR evaluation, all binders had a critical temperature of −12 °C based on the (S ≤ 300) MPa and (m ≥ 0.300) criteria. Thus, although diatomite and SBS modification altered the high- and intermediate-temperature rheological responses, they did not change the low-temperature critical-temperature classification under the adopted evaluation conditions. Diatomite modification increased binder consistency and viscosity and generally improved high-temperature rheological performance, but its effects were neither proportional to dosage nor identical for D1 and D2. D1 produced the most pronounced response in viscosity and some high-temperature indicators, whereas 5%SBS remained the strongest reference modifier in terms of high-temperature and post-aging rheological performance. The different responses of D1 and D2 may be associated with the morphological, elemental, and mineralogical characteristics identified by SEM–EDS, XRF, and XRD. However, because specific surface area and pore-size distribution were not measured, these relationships should be interpreted as possible differences in binder–mineral interaction rather than as direct effects of surface area or absorption capacity.

### 3.5. Indirect Tensile Strength

**Figure 12:**
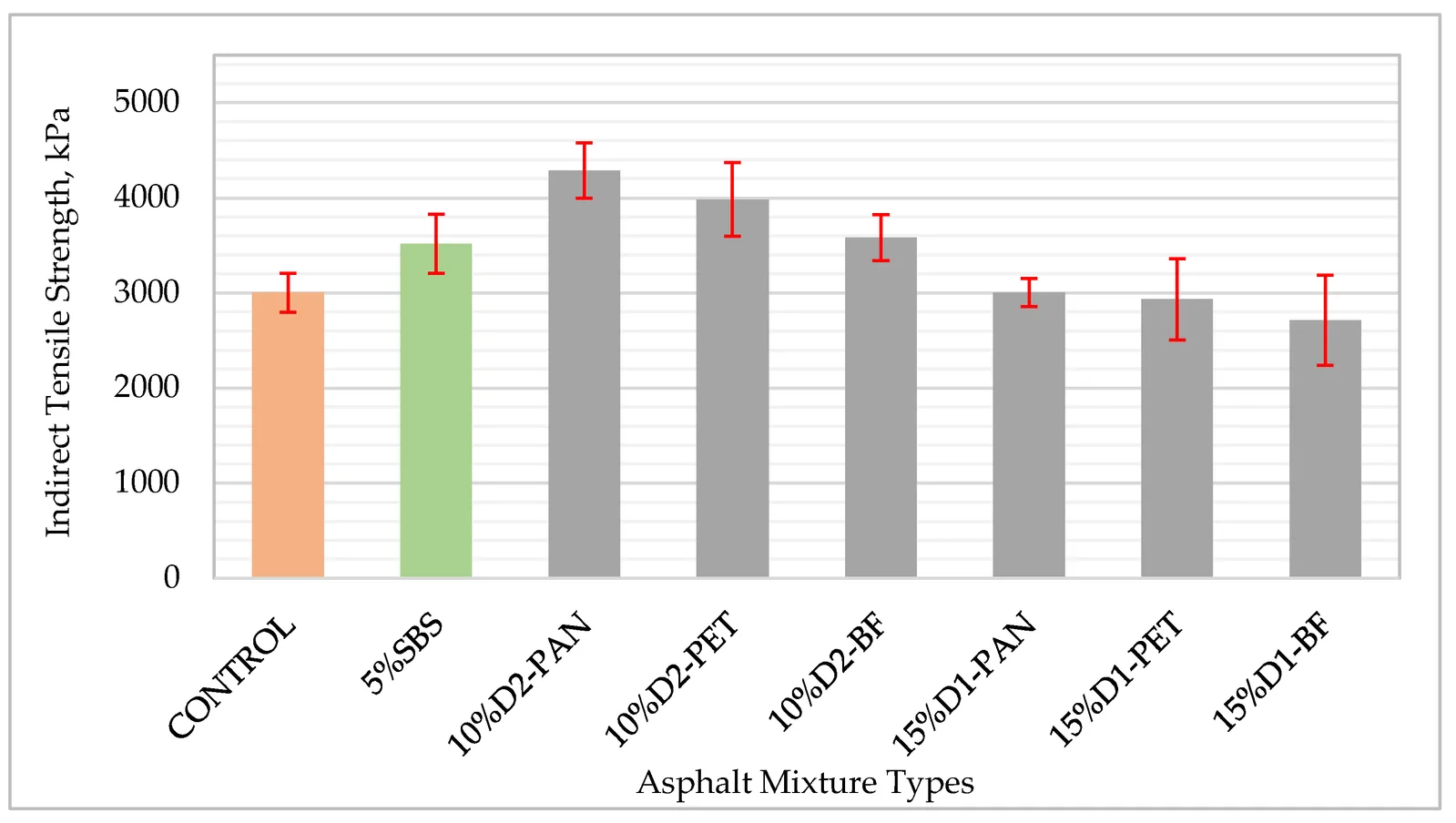
Indirect tensile strength of the asphalt mixtures at 0 °C. Values represent the mean ± standard deviation of three specimens per mixture. The orange and green bars represent the control and 5%SBS reference mixtures, respectively, while the gray bars represent the diatomite–fiber hybrid mixtures.

The indirect tensile strength (ITS) results obtained at 0 °C are presented in Figure 12. The unmodified control mixture exhibited an average ITS of approximately 3.0 MPa, whereas the 5% SBS-modified reference reached approximately 3.5 MPa. Among the hybrid mixtures, the highest average ITS values were obtained for the 10%D2 systems. The 10%D2-PAN mixture produced the highest value of approximately 4.29 MPa, followed by 10%D2-PET and 10%D2-BF at approximately 4.0 and 3.6 MPa, respectively. Thus, all three 10%D2 mixtures numerically exceeded the unmodified control and reached or surpassed the average ITS of the 5%SBS reference. In contrast, the 15%D1-PAN and 15%D1-PET mixtures produced values of approximately 3.0 MPa, while 15%D1-BF exhibited the lowest average value among the hybrid mixtures, at approximately 2.7 MPa.

The comparatively high ITS values of the 10%D2-PAN and 10%D2-PET mixtures may reflect the combined response of the diatomite-modified mastic and the reinforcing fibers. Diatomite can modify mastic consistency, while adequately dispersed fibers may contribute to local stress redistribution and restrict the development of tensile damage. The higher binder-retention ratios of PAN and PET and their locally embedded appearance in the SEM micrographs provide qualitative context for this interpretation. However, binder retention, interfacial adhesion, fiber dispersion, and stress transfer were not quantified within the mixtures. Therefore, these observations should be regarded as possible complementary mechanisms rather than direct evidence of synergistic reinforcement [83].

The lower average ITS values of the 15%D1 mixtures show that the response observed for the 10%D2 systems was not maintained across both diatomite formulations. Because diatomite type and dosage varied simultaneously between 10%D2 and 15%D1, their differences cannot be attributed independently to either diatomite source or content. Instead, the results represent the combined response of each complete diatomite–fiber system. Furthermore, ITS represents peak tensile strength under the applied test conditions and does not independently characterize fracture energy or post-peak crack propagation. The cracking response of the mixtures should therefore be evaluated together with the subsequent SCB results.

### 3.6. Evaluation of Moisture Susceptibility

**Figure 13:**
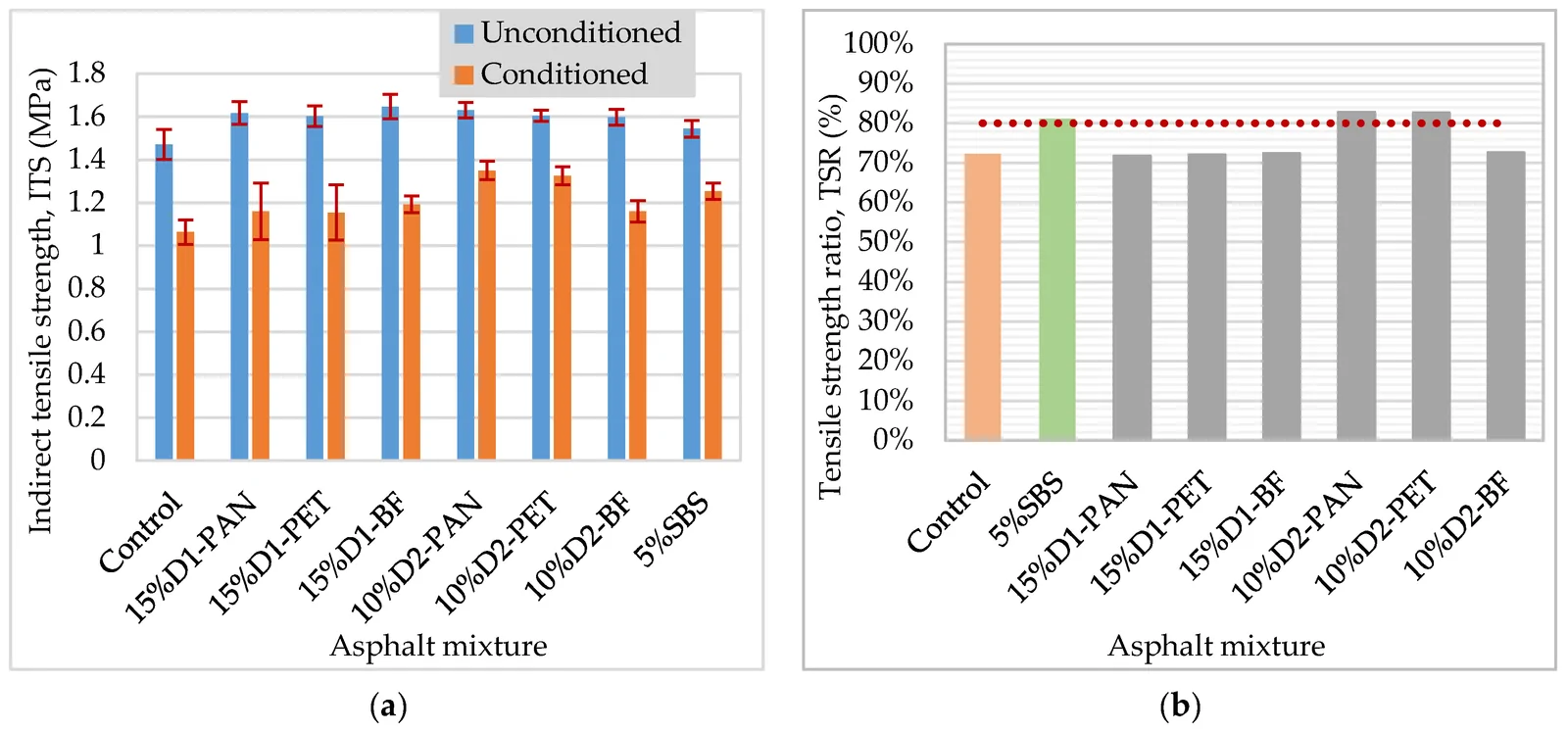
Moisture-susceptibility results of the asphalt mixtures: (**a**) dry and moisture-conditioned indirect tensile strength at 25 °C and (**b**) tensile strength ratio (TSR). Values in panel (**a**) represent the mean ± standard deviation of three specimens per conditioning group. The dashed line in panel (**b**) indicates the adopted minimum TSR criterion of 80%.

The dry and moisture-conditioned ITS values used to calculate the tensile strength ratio (TSR) are presented in Figure 13a. These specimens were tested at 25 °C and should not be directly compared with the independent ITS results obtained at 0 °C in Section 3.5. The strength retained after moisture conditioning varied among the mixtures. In particular, the 10%D2-PAN and 10%D2-PET mixtures maintained comparatively high proportions of their dry ITS values, whereas greater proportional strength losses were observed for 10%D2-BF and the three 15%D1 mixtures.

The corresponding TSR results are shown in Figure 13b, while the individual unconditioned and moisture-conditioned ITS measurements for all mixtures are provided in Table S7. The unmodified control exhibited a TSR of approximately 72%, whereas the 5% SBS-modified reference reached 81.13%. Among the hybrid mixtures, 10%D2-PAN and 10%D2-PET exhibited the highest mean TSR values, at 82.76% and 82.60%, respectively. For 10%D2-PAN, the unconditioned and conditioned ITS values were 1.632 ± 0.044 MPa and 1.350 ± 0.052 MPa, respectively; the corresponding values for 10%D2-PET were 1.605 ± 0.032 MPa and 1.326 ± 0.051 MPa (mean ± sample standard deviation; *n* = 3 per conditioning group). Although the mean TSR values of these two hybrid mixtures and the 5%SBS reference numerically exceeded the adopted 80% criterion, the margins were small, and the limited number of specimens does not establish statistically robust compliance with this threshold. These results should therefore be interpreted as preliminary evidence of favorable moisture-resistance performance under the investigated conditions rather than definitive confirmation of criterion satisfaction. In contrast, 10%D2-BF exhibited a TSR of approximately 73%, while the 15%D1-PAN, 15%D1-PET, and 15%D1-BF mixtures produced values of approximately 72%, below the adopted criterion. The comparatively high TSR values of 10%D2-PAN and 10%D2-PET may reflect the combined influence of the diatomite-modified mastic and the corresponding fiber type–dosage combinations on strength retention after moisture conditioning. Differences in binder retention, fiber dispersion, effective binder availability, and local interfacial morphology may have contributed to the observed response. However, these mechanisms were not directly quantified through surface-free-energy, contact-angle, water-diffusion, or direct-adhesion measurements. Moreover, diatomite type and content varied simultaneously between the 10%D2 and 15%D1 systems, and the PAN, PET, and basalt fibers were incorporated at different dosages. Therefore, the observed differences should be interpreted as the combined responses of the complete hybrid formulations rather than as independent effects of diatomite type, diatomite dosage, or fiber material.

### 3.7. Cantabro Particle-Loss Test Results

**Figure 14:**
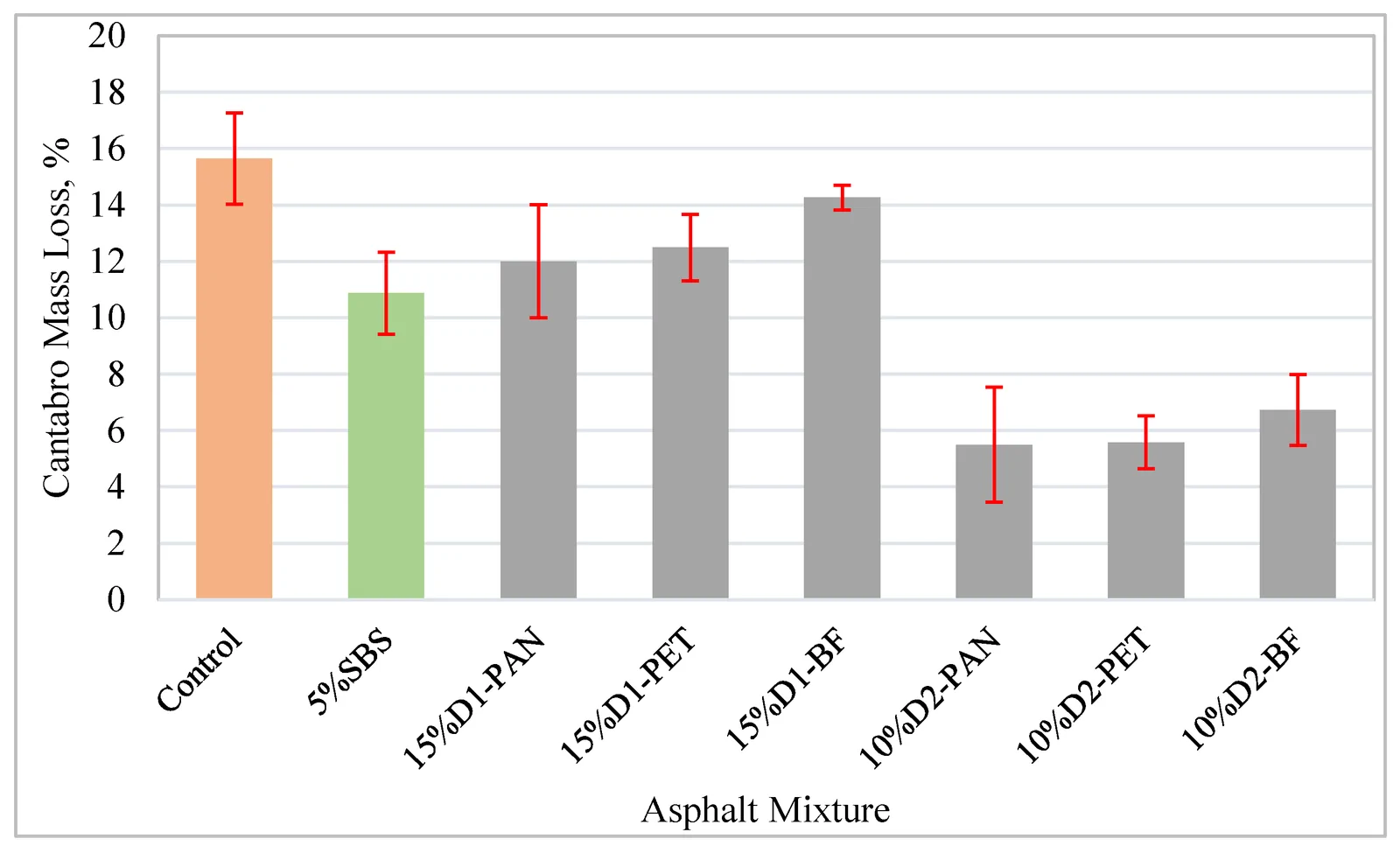
Cantabro loss of the asphalt mixtures at 25 °C. Values represent the mean ± standard deviation of three specimens per mixture.

The Cantabro loss results obtained at 25 °C are presented in Figure 14, with lower values indicating greater resistance to particle loss under the applied test conditions. The unmodified control exhibited an average loss of approximately 15.6%, which decreased to 10.8% for the 5% SBS-modified reference. The 15%D1-PAN, 15%D1-PET, and 15%D1-BF mixtures produced average losses of approximately 12.0%, 12.5%, and 14.2%, respectively. Although these values were lower than that of the control, they remained above the 5%SBS reference. In contrast, the 10%D2-PAN and 10%D2-PET mixtures exhibited the lowest average losses, at approximately 5.5% and 5.7%, respectively, followed by 10%D2-BF at approximately 6.7%. Thus, all three 10%D2 hybrid formulations numerically outperformed both the unmodified control and the 5%SBS reference in terms of particle-loss resistance.

The comparatively low Cantabro losses of the 10%D2 formulations may reflect the combined contributions of the diatomite-modified mastic and the corresponding fiber type–dosage combinations to mixture cohesion. Cantabro response can be influenced by binder–aggregate interaction, mastic cohesion, air-void structure, effective binder availability, and fiber dispersion. Properly distributed fibers may help restrict particle separation and preserve mixture integrity during impact and abrasion, while the diatomite-modified binder may alter the consistency and internal structure of the mastic [85,86]. The lower losses obtained for the PAN- and PET-containing mixtures than for the corresponding basalt-fiber mixtures are also qualitatively consistent with their higher binder-retention ratios. However, these observations do not directly establish the mechanisms responsible for the measured Cantabro response.

The differences between the 10%D2 and 15%D1 groups cannot be attributed independently to diatomite type or dosage because both factors varied simultaneously. Similarly, the PAN, PET, and basalt fibers were incorporated at different dosages; consequently, comparisons among them represent the responses of the complete fiber type–dosage formulations rather than the isolated effect of fiber material. Moreover, because the Cantabro procedure was originally developed primarily for open-graded asphalt mixtures, its application to the dense-graded mixtures investigated here was used for relative comparison under identical specimen geometry and testing conditions rather than for assessment against a universal acceptance threshold [87].

### 3.8. Repeated-Load Creep Test Results

**Figure 15:**
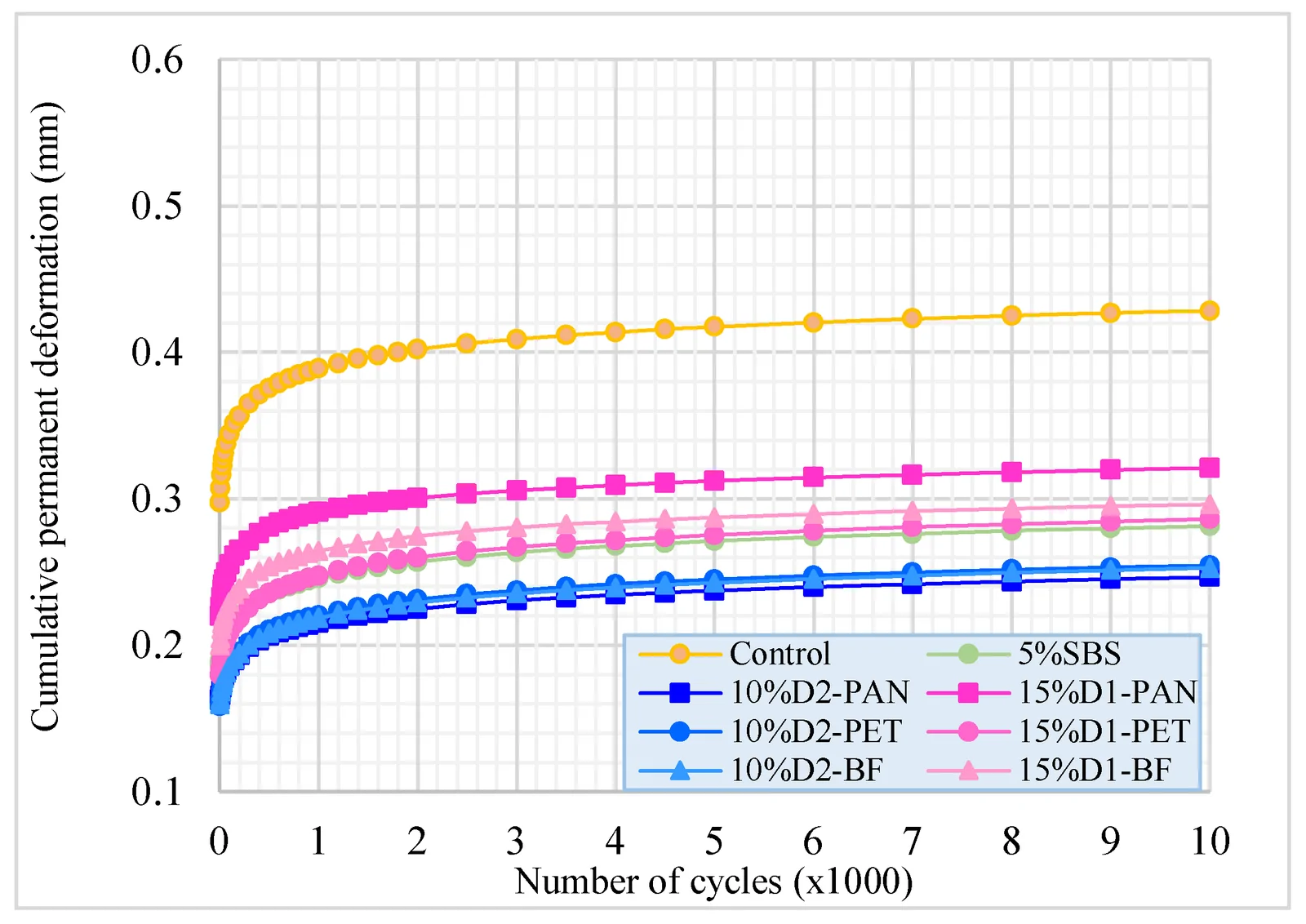
Mean cumulative permanent-deformation curves of the asphalt mixtures during the repeated-load creep test.

The mean cumulative permanent-deformation curves obtained at 40 °C are presented in Figure 15. All mixtures exhibited rapid deformation accumulation during the initial loading stage, followed by a progressively lower accumulation rate as the number of cycles increased. At the end of 10,000 cycles, the unmodified control exhibited the highest average permanent deformation, at approximately 0.43 mm. The corresponding value decreased to approximately 0.28 mm for the 5% SBS-modified reference. All diatomite–fiber hybrid formulations produced lower average final deformation than the control under the applied test conditions.

After 10,000 loading cycles, the unmodified control exhibited the highest mean cumulative permanent deformation of 0.428 ± 0.166 mm, whereas the 5% SBS-modified mixture showed a lower value of 0.282 ± 0.056 mm. Among the hybrid formulations, the 10%D2-PAN, 10%D2-PET, and 10%D2-BF mixtures exhibited mean cumulative permanent deformations of 0.247 ± 0.054, 0.255 ± 0.021, and 0.253 ± 0.039 mm, respectively. The corresponding values for the 15%D1-PAN, 15%D1-PET, and 15%D1-BF mixtures were 0.321 ± 0.060, 0.286 ± 0.046, and 0.296 ± 0.034 mm, respectively.

The reduced deformation observed in the hybrid formulations may reflect the combined influence of the diatomite-modified mastic and fiber reinforcement on the accumulation of axial deformation. Changes in mastic consistency, fiber dispersion, and local fiber–mastic interaction may contribute to resistance under repeated loading [65,67,88]. However, these mechanisms were not directly quantified by the creep test. In addition, diatomite type and dosage varied simultaneously between the 10%D2 and 15%D1 systems, while the PAN, PET, and basalt fibers were incorporated at different dosages. Consequently, the results should be interpreted as the responses of the complete hybrid formulations under the applied testing conditions. The small numerical differences among the 10%D2 formulations should not be considered evidence of a definitive performance ranking without additional statistical support.

### 3.9. Semi-Circular Bending Test Results

**Figure 16:**
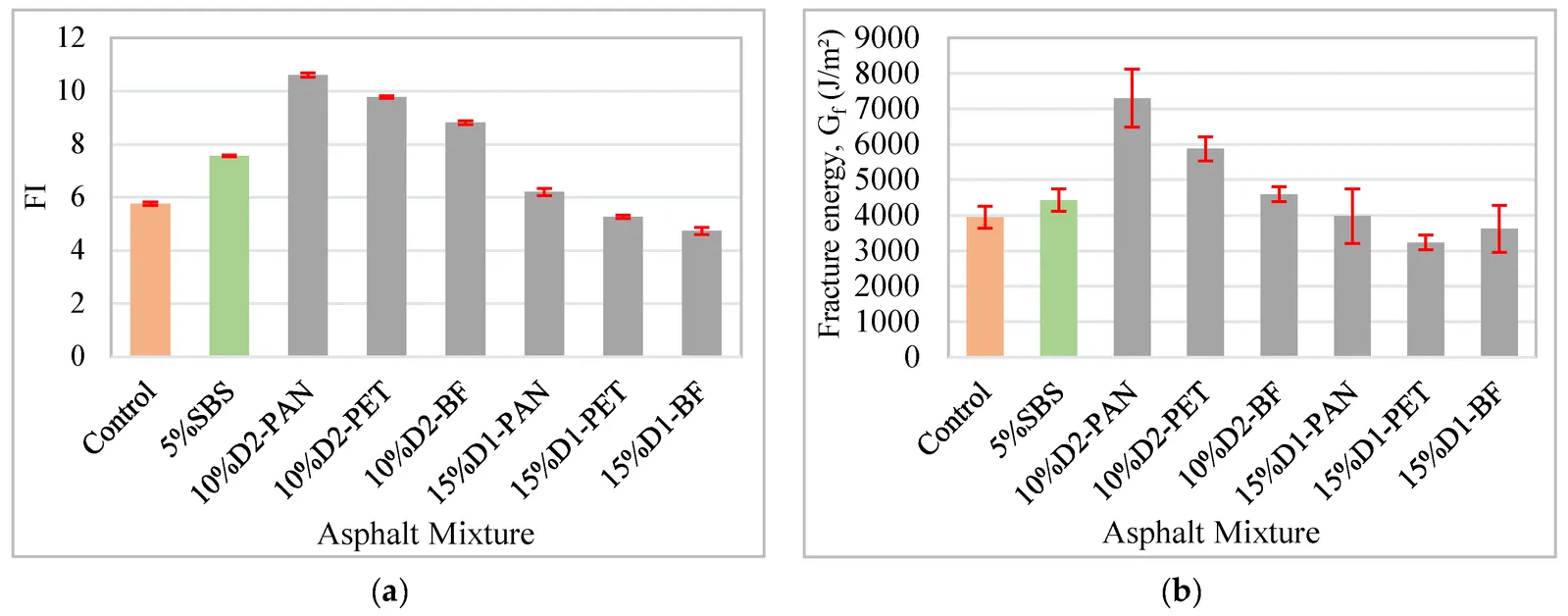
SCB fracture parameters of the asphalt mixtures: (**a**) flexibility index and (**b**) fracture energy. Values represent the mean ± standard deviation of six SCB specimens per mixture.

The flexibility index (FI) and fracture energy (G_f_) results are presented in Figure 16a and Figure 16b, respectively. Because the SCB specimens had a diameter of 100 mm rather than the nominal 150 mm geometry specified in AASHTO TP 124-18 [68], specimen size may have affected the absolute magnitudes of both parameters [72]. Accordingly, the reported FI and G_f_ values were used for within-study comparisons and should not be considered directly interchangeable with values obtained using the standardized 150 mm configuration.

As shown in Figure 16a, the unmodified control and 5% SBS-modified reference exhibited average FI values of approximately 5.7 and 7.5, respectively. Among the hybrid formulations, 10%D2-PAN produced the highest average FI, at approximately 10.6, followed by 10%D2-PET and 10%D2-BF at approximately 9.7 and 8.8, respectively. All three 10%D2 formulations therefore numerically exceeded both reference mixtures. In contrast, the 15%D1-PAN, 15%D1-PET, and 15%D1-BF mixtures exhibited average FI values of approximately 6.2, 5.3, and 4.7, respectively. Because FI combines fracture energy with the post-peak slope of the load–displacement curve, higher values indicate comparatively greater tolerance to post-peak crack propagation under the applied test conditions [89].

The fracture-energy results in Figure 16b provide complementary information on the energy absorbed during fracture. The control and 5%SBS mixtures produced average G_f_ values of approximately 3.9 and 4.4 kJ/m^2^, respectively. The highest average G_f_ was obtained for 10%D2-PAN, at approximately 7.3 kJ/m^2^, followed by 10%D2-PET and 10%D2-BF at approximately 5.8 and 4.6 kJ/m^2^, respectively. The corresponding values for 15%D1-PAN, 15%D1-PET, and 15%D1-BF were approximately 4.0, 3.2, and 3.6 kJ/m^2^. Thus, 10%D2-PAN exhibited the highest average values of both FI and G_f_, indicating a comparatively high energy-absorption capacity and tolerance to post-peak crack propagation within the adopted geometry. Nevertheless, FI and G_f_ should not be treated as equivalent performance indicators because FI also incorporates the post-peak slope. The PAN > PET > BF order observed for both parameters in the 10%D2 group was not fully reproduced by the Gf results of the 15%D1 group.

The differences between the 10%D2 and 15%D1 groups cannot be attributed independently to diatomite type or content because both factors varied simultaneously. Likewise, PAN, PET, and basalt fibers were incorporated at different dosages; therefore, comparisons among them represent the responses of the complete fiber type–dosage formulations. Although fiber bridging, pull-out, dispersion, and interfacial interaction may have contributed to the observed fracture response, these mechanisms were not directly quantified. The SCB results should therefore be interpreted as comparative evidence of the performance of the investigated hybrid formulations under identical specimen geometry and testing conditions rather than as proof of individual material mechanisms.

## 4. Conclusions

The combined evaluation of modifier characteristics, binder rheology, and mixture performance showed that the benefits of diatomite–microfiber modification depended on the formulation and the performance criterion considered. Physicochemical characterization helped interpret the observed behavior, although it did not establish direct links between individual material properties and mixture performance.

Diatomite addition generally increased binder consistency and improved the high-temperature rheological response. These changes were not proportional to diatomite content, and the measured low-temperature BBR classification remained unchanged. The 5%SBS binder exhibited the highest DSR-based high-temperature critical temperatures among the investigated binders, both before and after RTFOT aging. However, the trends observed in the binder tests were not fully reflected in the mixture tests, showing the importance of evaluating both scales. Higher tensile strength also did not necessarily coincide with better moisture resistance.

Of the mixtures tested, 10%D2-PAN showed a favorable overall combination of responses. It had the highest mean indirect tensile strength and SCB fracture indicators, together with relatively low Cantabro loss and permanent deformation. The 10%D2-PET mixture also performed favorably across several tests. Both mixtures had mean TSR values slightly above the adopted 80% criterion, although their proximity to this threshold and the limited number of specimens warrant further verification of moisture resistance. These findings support further investigation of the two formulations.

Because diatomite source and content, as well as fiber type and dosage, were not varied independently at the mixture scale, the comparisons describe complete formulations rather than the separate contributions of their constituents. The findings remain descriptive and specific to the investigated materials and laboratory conditions, with the adapted 100 mm SCB results intended for within-study comparisons. Further validation is needed before extending these conclusions to other material combinations or practical applications.

## 5. Recommendations and Limitations

The findings are limited to the investigated material sources, modifier contents, fiber formulations, and laboratory conditions. Because D1 and D2 were evaluated at different mixture-level contents (15%D1 and 10%D2), the effects of diatomite source and dosage cannot be independently separated. Similarly, PAN, PET, and basalt fibers were incorporated at different dosages; therefore, their responses represent complete fiber type–dosage formulations rather than isolated fiber-material effects. The optimum binder content was determined before fiber incorporation and was not independently re-optimized for each fiber-reinforced mixture. Differences in fiber-specific binder retention and effective binder availability may consequently have contributed to the measured performance. Moreover, the comparisons were interpreted descriptively and do not demonstrate statistical significance. SEM–EDS, XRF, and XRD provided qualitative physicochemical evidence but did not directly quantify surface area, interfacial adhesion, fiber dispersion, or load-transfer efficiency. The FI and G_f_ values obtained using the adapted 100 mm SCB geometry should also be used primarily for within-study comparison.

Future studies should evaluate D1 and D2 at common dosage levels using a factorial design and independently optimize binder content and fiber dosage for each formulation. Quantitative surface and interfacial characterization would help clarify the mechanisms linking diatomite and fiber properties to mixture performance. The observed trends should also be verified using the standardized SCB geometry, complementary rutting and fatigue tests, additional aging and moisture-conditioning procedures, and a broader range of binder and aggregate sources. Field-scale validation, together with economic and environmental assessment, is recommended before practical implementation.

## Figures and Tables

**Table 1:** Properties of the base binder.

Test	Result	Specification Limits	Test Method
Specific gravity (25 °C)	1.045	1.0–1.1	TS EN 15326+A1 [17]
Softening point (°C)	49.83	46–54	TS EN 1427 [18]
Flash point (°C)	235	≥230	TS EN ISO 2592 [19]
Penetration (25 °C) 0.1 mm	60.16	50–70	TS EN 1426 [20]
Solubility (%)	99.2	≥99	TS EN 12592 [21]

**Table 2:** Aggregate properties.

Properties	Test Result/Specification	Test Standard
Polished stone value	57.9/≥50 (PSV_50_)	TS EN 1097-8 [22]
Soundness (MgSO_4_) (%)	1.25/≤16 (MS_16_)	TS EN 1367-2 [23]
Los Angeles abrasion loss (%)	12/≤27 (LA_27_)	AASHTO T-96 [24]
Flakiness index (%)	11/≤25 (FI_25_)	BS 812-105.1 [25]
Water absorption (%)	0.7/≤2.0 (WA_24_ 2.0)	TS EN 1097-6 [26]
Micro-Deval abrasion loss (%)	17/≤20 (M_DE_ 20)	TS EN 1097-1 [27]
Methylene Blue (%)	1/≤1.5 (MB1.5); ≤3.0 (MB3.0)	TS EN 933-9 [28]

**Table 3:** Physical and mechanical properties of PAN, PET, and basalt microfibers.

Microfibers	Length (mm)	Diameter (μm)	Density (g/cm^3^)	Tensile Stress (MPa)	Melting Point (°C)
PAN	6–12	13 ± 3	1.18	600–900	210
PET	6 ± 1.5	20 ± 5	1.36 ± 0.04	>700	260
Basalt	6–12	7–30	2.8	1050–3500	600

**Table 4:** General properties of the SBS polymer [37].

Property	Standard	Value
Molecular structure	–	Linear
Styrene/butadiene ratio	–	29/71
Specific gravity	ISO 2781 [38]	0.94
Tensile strength (MPa)	ISO 37 [39]	33
Hardness (Shore A)	ASTM D 2240 [40]	70
300% modulus (MPa)	ISO 37 [39]	4.8
Melt flow rate (g/10 min)	ISO 1133 [41]	<1
Elongation at break (%)	ISO 37 [39]	1000

**Table 5:** Mixture formulations and Marshall design parameters of the binder systems used in the mixture-level performance evaluation.

Parameter	Control	5%SBS	15%D1	10%D2	Specification Limit [16]
Associated mixture formulation(s)	Control	5%SBS	15%D1-PAN; 15%D1-PET; 15%D1-BF	10%D2-PAN; 10%D2-PET; 10%D2-BF	–
Modifier content (% by base-binder mass)	–	5	15	10	–
OBC (%)	5.88	6.35	6.24	6.04	4–7
Marshall stability (kg)	1805	1728	1612.59	1687.01	≥900
Flow (mm)	3.34	3.84	4	3.95	2–4
Voids filled with asphalt, VFA (%)	74.36	74.8	76.18	74.9	65–75
Voids in mineral aggregate, VMA (%)	15.78	15.96	16.34	15.98	14–16
Filler-to-binder ratio	0.83	0.77	0.79	0.81	≤1.5

**Table 6:** Local semi-quantitative EDS compositions of the D1 and D2 diatomites.

	D1 Diatomite		D2 Diatomite	
Element	Atomic Percentage (at.%)	Weight Percentage (wt.%)	Atomic Percentage (at.%)	Weight Percentage (wt.%)
C	37.8	27.9	7.8	5
O	48.6	47.8	67	56.9
Mg	0.5	0.8	0.7	0.9
Al	1.4	2.3	1.7	2.5
Si	10.4	18.0	22.1	32.9
K	0.1	0.3	0.1	0.3
Ca	0.8	2.0	0.4	0.7
Fe	0.2	0.8	0.3	0.8

**Table 7:** Local semi-quantitative EDS compositions of the PAN, PET, and basalt fibers.

Fiber	Main Elemental Composition (wt.%)
PAN	C 54.7; N 38.1; O 5.5
PET	C 47.5; O 52.0
Basalt	Si 52.2; Al 15.4; Fe 14.6; Ca 10.4; K 4.0; Mg 3.4

**Table 8:** Major oxide compositions of the D1 and D2 diatomites determined by XRF.

Oxide	D1 (wt.%)	D2 (wt.%)
SiO_2_	72.1	69.2
Al_2_O_3_	9.02	9.47
Fe_2_O_3_	3.31	3.15
MgO	3.01	3.02
CaO	2.30	2.50
K_2_O	1.22	1.33

**Table 9:** Conventional physical properties of the unmodified and modified binders.

Property	Control	10%D1	15%D1	10%D2	15%D2	5%SBS
Penetration (0.1 mm)	60.16	54.24	57.23	59.23	45.17	39.99
Softening point (°C)	49.83	52.65	53.13	55.30	54.68	67.08
Penetration index	−0.807	−0.364	−0.120	0.469	−0.333	1.782
Specific gravity	1.045	1.119	1.099	1.082	1.086	1.014

**Table 10:** Rheological and aging characteristics of the unmodified and modified binders.

Test/Parameter	Control	10%D1	15%D1	10%D2	15%D2	5%SBS
Dynamic shear rheometer (DSR) Critical temperature (G*/sin δ > 1 kPa), °C	67.2	70.5	69.6	69.1	67.8	82.5
Rolling thin-film oven test (RTFOT)						
Mass change (%)	0.1	0.2	0.3	0.3	0.2	0.05
Increase in softening point (°C)	0.2	3.2	5.5	4.2	5.5	0.9
Retained penetration (%)	76	87	70	74	84	85
Critical temperature (G*/sin δ ≥ 2.2 kPa) (°C)	66.3	68.9	69.3	68.1	66.1	79
RTFOT + PAV-aged binders						
DSR critical temperature (G*·sin δ ≤ 5000 kPa) (°C)	28.2	30.9	29.6	28.8	27.1	24.2
BBR critical temperature (S ≤ 300 MPa and m ≥ 0.300) (°C)	−12	−12	−12	−12	−12	−12

## Data Availability

The original contributions presented in this study are included in the article. Further inquiries can be directed to the corresponding author.

## Supplementary Materials

The following supporting information can be downloaded at: https://www.mdpi.com/article/10.3390/polym18202597/s1: Figure S1: SEM images of D1 diatomite at different magnifications: (a) 100×, (b) 1000×, (c) 2500×, and (d) 10,000×; Figure S2: SEM images of D2 diatomite at different magnifications: (a) 100×, (b) 1000×, (c) 2500×, and (d) 10,000×; Figure S3: EDS spectra of the diatomites: (a) D1 and (b) D2; Figure S4: SEM images of the basalt filler at different magnifications: (a) 250×, (b) 1000×, (c) 2500×, and (d) 10,000×; Figure S5: EDS characterization of the basalt filler: (a) combined elemental map and (b) EDS spectrum; Figure S6: SEM images of the PAN fiber at different magnifications: (a) 250×, (b) 300×, (c) 500×, and (d) 3000×; Figure S7: SEM images of the PET fiber: (a) 200×, (b) 400×, (c) 3000×, and (d) fiber diameter; Figure S8: SEM images of the basalt fiber at different magnifications: (a) 250×, (b) 500×, (c) 600×, and (d) 3000×; Figure S9: EDS spectra of the microfibers; Figure S10: XRF spectra of the diatomites; Figure S11: XRF spectrum of the basalt filler; Figure S12: XRD pattern of the basalt filler; Figure S13: SEM images of the 15%D1-PAN fiber-reinforced asphalt mixture at different magnifications: (a) 100×, (b) 250×, (c) 1000×, and (d) 5000×; Figure S14: SEM images of the 15%D1-PET fiber-reinforced asphalt mixture at different magnifications: (a) 100×, (b) 250×, (c) 1000×, and (d) 5000×; Figure S15: SEM images of the 15%D1-BF fiber-reinforced asphalt mixture at different magnifications: (a) 100×, (b) 250×, (c) 1000×, and (d) 5000×; Figure S16: SEM images of the 10%D2-PAN fiber-reinforced asphalt mixture at different magnifications: (a) 100×, (b) 250×, (c) 1000×, and (d) 5000×; Figure S17: SEM images of the 10%D2-PET fiber-reinforced asphalt mixture at different magnifications: (a) 100×, (b) 250×, (c) 1000×, and (d) 5000×; Figure S18: SEM images of the 10%D2-BF fiber-reinforced asphalt mixture at different magnifications: (a) 100×, (b) 250×, (c) 1000×, and (d) 5000×; Figure S19: SEM images of the unmodified control asphalt mixture; Figure S20: SEM images of the 5% SBS-modified asphalt mixture; Table S1: Elemental composition of the basalt filler determined by EDS analysis; Table S2: Elemental compositions of the PAN, PET, and basalt fibers determined by EDS analysis; Table S3: Elemental composition of D1 and D2 diatomites determined by XRF analysis; Table S4: Oxide composition of D1 and D2 diatomites determined by XRF analysis; Table S5: Elemental composition of the basalt filler determined by XRF analysis; Table S6: Oxide composition of the basalt filler determined by XRF analysis; Table S7: Individual unconditioned and moisture-conditioned ITS measurements, mean values, standard deviations, and TSR results for all asphalt mixtures.
