# Supplementary material for: Physicochemical Characterization, Binder Rheology, and Engineering Performance of Diatomite–Microfiber Hybrid Asphalt Systems
Source: Polymers (Basel). 2026 Oct 21;18(20):2597. doi: 10.3390/polym18202597 (PMC13708819; doi:10.3390/polym18202597)
Supplement: Supplementary file 1 [file polymers-18-02597-s001.zip › polymers-4619043-supplementary.pdf]

# Supporting Information

## Physicochemical Characterization, Binder Rheology, and Engineering Performance of Diatomite–Microfiber Hybrid Asphalt Systems

**Mustafa Taha Aslan [1,*], Erol İskender [2]**

[1] Graduate School of Natural and Applied Sciences, Karadeniz Technical University, Trabzon 61080, Türkiye.
[2] Department of Civil Engineering, Faculty of Technology, Karadeniz Technical University, Trabzon 61830, Türkiye; eiskender@ktu.edu.tr (E.I.)
* Correspondence: aslanmustafataha@gmail.com

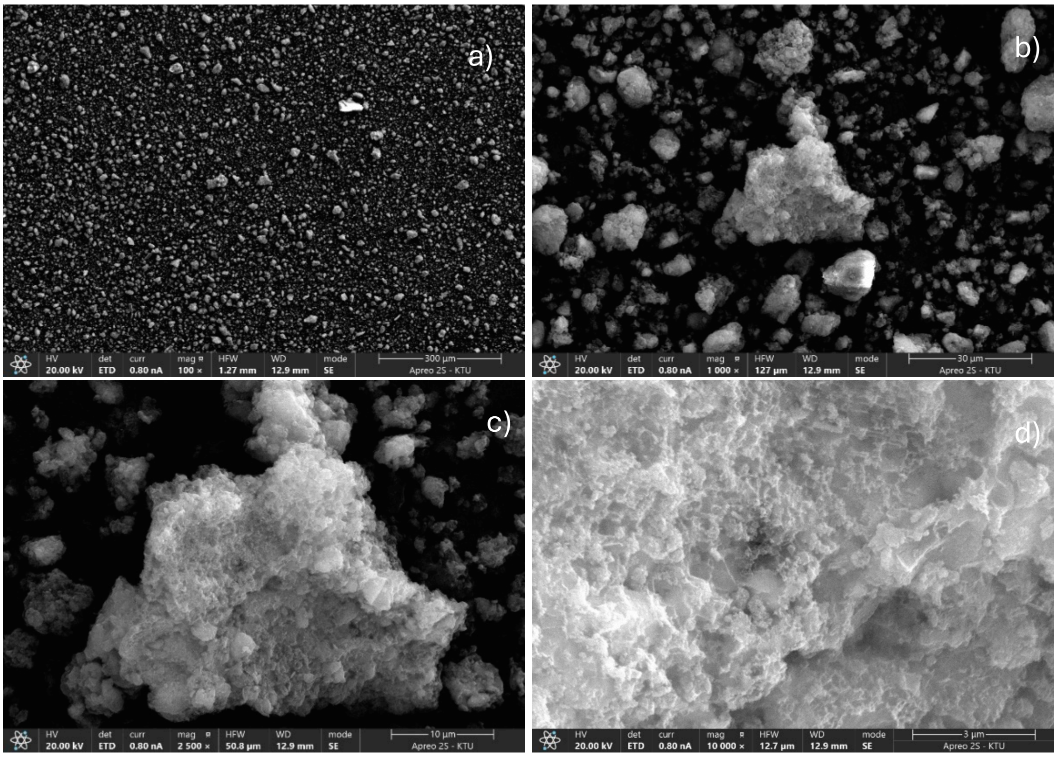


**Figure S1.** SEM images of D1 diatomite at different magnifications: (a) 100×, (b) 1,000×, (c) 2,500×, and (d) 10,000×.

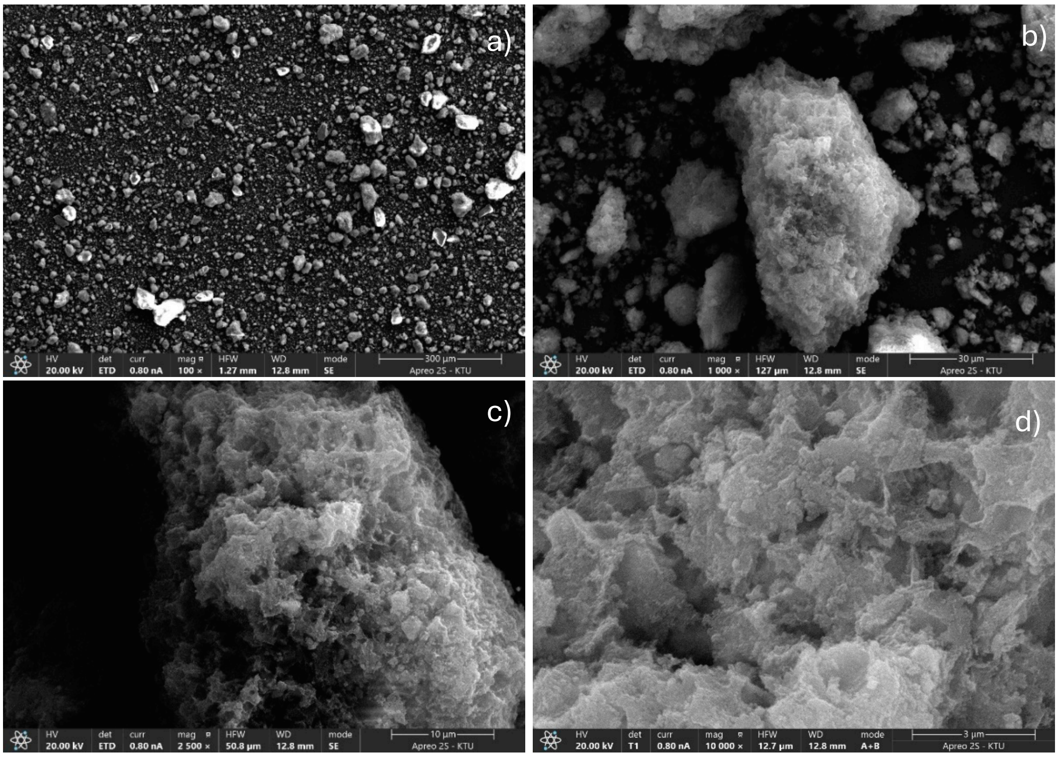


**Figure S2.** SEM images of D2 diatomite at different magnifications: (a) 100×, (b) 1,000×, (c) 2,500×, and (d) 10,000×.

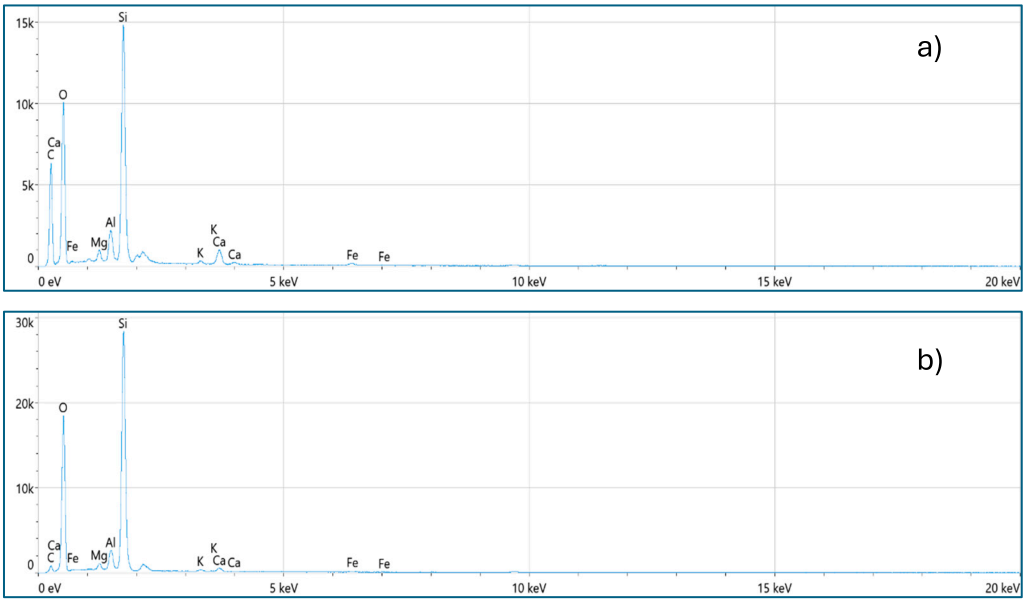


**Figure S3.** 1 EDS spectra of the diatomites: (a) D1 and (b) D2.

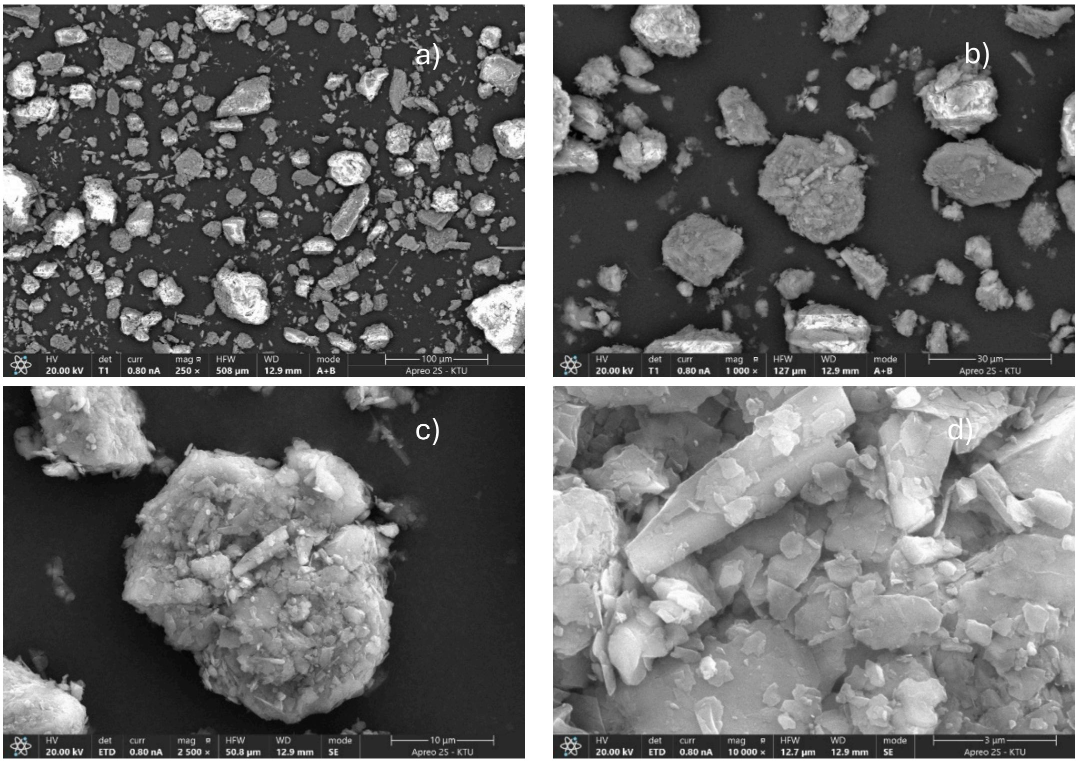


**Figure S4.** SEM images of the basalt filler at different magnifications: (a) 250×, (b) 1,000×, (c) 2,500×, and (d) 10,000×.

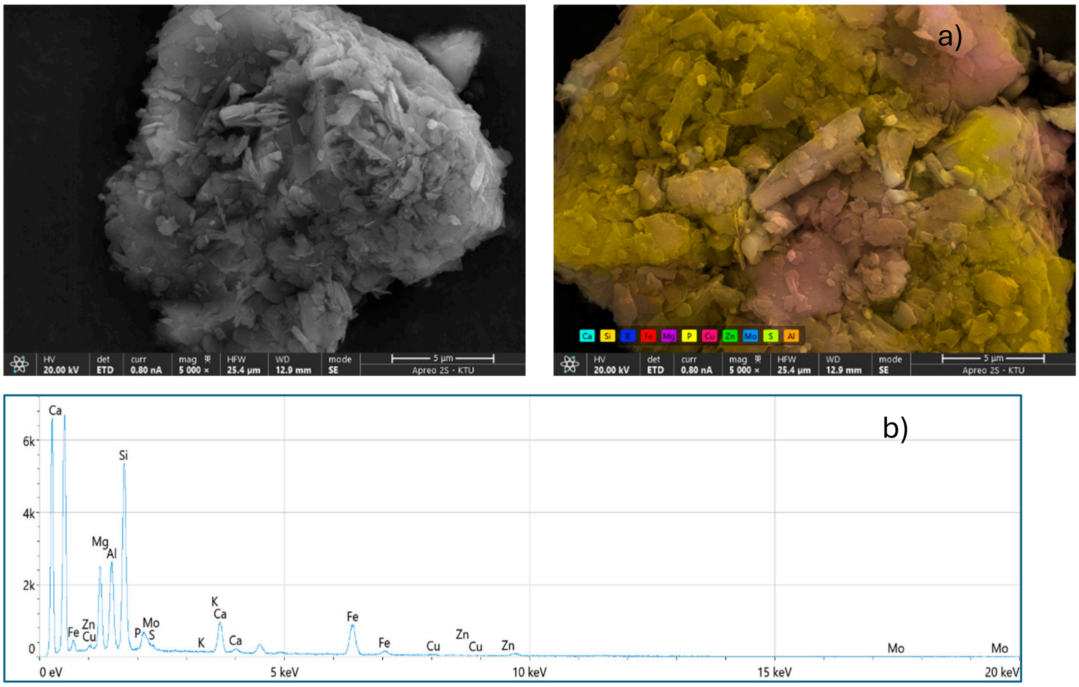


**Figure S5.** EDS characterization of the basalt filler: (a) combined elemental map and (b) EDS spectrum.

**Table S1.** Elemental composition of the basalt filler determined by EDS analysis.

| Element | Atomic percentage (at.%) | Weight percentage (wt.%) |
|---|---|---|
| Si | 42.3 | 37.1 |
| Mg | 18.6 | 14.1 |
| Al | 17.8 | 15.0 |
| Fe | 13.8 | 24 |
| Ca | 6.9 | 8.6 |
| Cu | 0.5 | 1.1 |

**Table S2.** Elemental compositions of the PAN, PET, and basalt fibers determined by EDS analysis.

| Fiber | Element | Atomic percentage (at.%) | Weight percentage (wt.%) |
|---|---|---|---|
| PAN | C | 59.3 | 54.7 |
| | N | 35.4 | 38.1 |
| | O | 4.5 | 5.5 |
| | Si | 0.6 | 1.3 |
| | Ca | 0.1 | 0.2 |
| | K | <DL | 0.1 |
| PET | C | 54.7 | 47.5 |
| | O | 45 | 52 |
| | Al | 0.2 | 0.3 |
| | Si | 0.1 | 0.2 |
| | Ca | <DL | <DL |
| | Fe | <DL | 0.1 |
| BF | Si | 58.2 | 52.2 |
| | Al | 17.9 | 15.4 |
| | Ca | 8.2 | 10.4 |
| | Fe | 8.2 | 14.6 |
| | Mg | 4.3 | 3.4 |
| | K | 3.2 | 4 |

**Table S3.** Elemental composition of D1 and D2 diatomites determined by XRF analysis.

| D1 diatomite | | | D2 diatomite | | |
|---|---|---|---|---|---|
| Element | Symbol | Content (wt.%) | Element | Symbol | Content (wt.%) |
| Oxygen | O | 57.3 | Oxygen | O | 57 |
| Silicon | Si | 29.9 | Silicon | Si | 29 |
| Aluminum | Al | 4.33 | Aluminum | Al | 4.58 |
| Iron | Fe | 1.90 | Iron | Fe | 1.84 |
| Carbon | C | 1.86 | Carbon | C | 1.76 |
| Magnesium | Mg | 1.67 | Magnesium | Mg | 1.69 |
| Calcium | Ca | 1.38 | Calcium | Ca | 1.52 |
| Potassium | K | 0.856 | Potassium | K | 0.940 |
| Titanium | Ti | 0.202 | Boron | B | 0.701 |
| Sodium | Na | 0.175 | Titanium | Ti | 0.268 |
| Phosphorus | P | 0.138 | Sodium | Na | 0.236 |
| Manganese | Mn | 0.0929 | Fluorine | F | 0.144 |
| Barium | Ba | 0.0546 | Phosphorus | P | 0.139 |
| Vanadium | V | 0.0318 | Manganese | Mn | 0.114 |
| Strontium | Sr | 0.0172 | Barium | Ba | 0.0427 |
| Rubidium | Rb | 0.0096 | Strontium | Sr | 0.0310 |
| Sulphur | S | 0.0075 | Chromium | Cr | 0.0133 |
| Chromium | Cr | 0.0070 | Rubidium | Rb | 0.0095 |
| Zinc | Zn | 0.0053 | Zirconium | Zr | 0.0090 |
| Zirconium | Zr | 0.0051 | Sulfur | S | 0.0076 |
| Nickel | Ni | 0.0050 | Zinc | Zn | 0.0048 |

**Table S4.** Oxide composition of D1 and D2 diatomites determined by XRF analysis.

| D1 diatomite | | | D2 diatomite | | |
|---|---|---|---|---|---|
| Oxide component | Chemical formula | Content (wt.%) | Oxide component | Chemical formula | Content (wt.%) |
| Silicon dioxide | $SiO_2$ | 72.1 | Silicon dioxide | $SiO_2$ | 69.2 |
| Aluminum oxide | $Al_2O_3$ | 9.02 | Aluminum oxide | $Al_2O_3$ | 9.47 |
| Carbon dioxide | $CO_2$ | 7.62 | Carbon dioxide | $CO_2$ | 7.16 |
| Iron(III) oxide | $Fe_2O_3$ | 3.31 | Iron(III) oxide | $Fe_2O_3$ | 3.15 |
| Magnesium oxide | MgO | 3.01 | Magnesium oxide | MgO | 3.02 |
| Calcium oxide | CaO | 2.30 | Calcium oxide | CaO | 2.50 |
| Potassium oxide | $K_2O$ | 1.22 | Boron (III) oxide | $B_2O_3$ | 2.42 |
| Titanium dioxide | $TiO_2$ | 0.399 | Potassium oxide | $K_2O$ | 1.33 |
| Phosphorus pentoxide | $P_2O_5$ | 0.374 | Titanium dioxide | $TiO_2$ | 0.531 |
| Sodium oxide | $Na_2O$ | 0.254 | Phosphorus pentoxide | $P_2O_5$ | 0.373 |
| Manganese oxide | MnO | 0.146 | Sodium oxide | $Na_2O$ | 0.342 |
| Barium oxide | BaO | 0.0734 | Manganese oxide | MnO | 0.176 |
| Vanadium(V) oxide | $V_2O_5$ | 0.0680 | Fluorine | F | 0.152 |
| Strontium oxide | SrO | 0.0252 | Barium oxide | BaO | 0.0566 |
| Sulfur trioxide | $SO_3$ | 0.0221 | Strontium oxide | SrO | 0.0446 |
| Rubidium oxide | $Rb_2O$ | 0.0130 | Chromium(III) oxide | $Cr_2O_3$ | 0.0231 |
| Chromium(III) oxide | $Cr_2O_3$ | 0.0121 | Sulfur trioxide | $SO_3$ | 0.0222 |
| Zirconium oxide | $ZrO_2$ | 0.0082 | Zirconium oxide | $ZrO_2$ | 0.0147 |
| Zinc oxide | ZnO | 0.0081 | Rubidium oxide | $Rb_2O$ | 0.0126 |
| Nickel oxide | NiO | 0.0079 | Zinc oxide | ZnO | 0.0073 |

**Table S5.** Elemental composition of the basalt filler determined by XRF analysis.

| Element | Symbol | Content (wt.%) |
|---|---|---|
| Oxygen | O | 52.1 |
| Silicon | Si | 19.6 |
| Iron | Fe | 7.85 |
| Aluminium | Al | 6.98 |
| Calcium | Ca | 4.66 |
| Magnesium | Mg | 4.64 |
| Carbon | C | 1.47 |
| Sodium | Na | 1.46 |
| Titanium | Ti | 0.80 |
| Phosphorus | P | 0.16 |
| Potassium | K | 0.15 |
| Manganese | Mn | 0.15 |

**Table S6.** Oxide composition of the basalt filler determined by XRF analysis.

| Oxide component | Chemical formula | Content (wt.%) |
|---|---|---|
| Silicon dioxide | $SiO_2$ | 46.3 |
| Aluminium oxide | $Al_2O_3$ | 14.4 |
| Iron(III) oxide | $Fe_2O_3$ | 13.2 |
| Magnesium oxide | MgO | 8.30 |
| Calcium oxide | CaO | 7.45 |
| Carbon dioxide | $CO_2$ | 5.84 |
| Sodium oxide | $Na_2O$ | 2.11 |
| Titanium dioxide | $TiO_2$ | 1.54 |
| Phosphorus pentoxide | $P_2O_5$ | 0.41 |
| Manganese oxide | MnO | 0.22 |
| Potassium oxide | $K_2O$ | 0.21 |
| Strontium oxide | SrO | 0.04 |
| Sulphur trioxide | $SO_3$ | 0.03 |

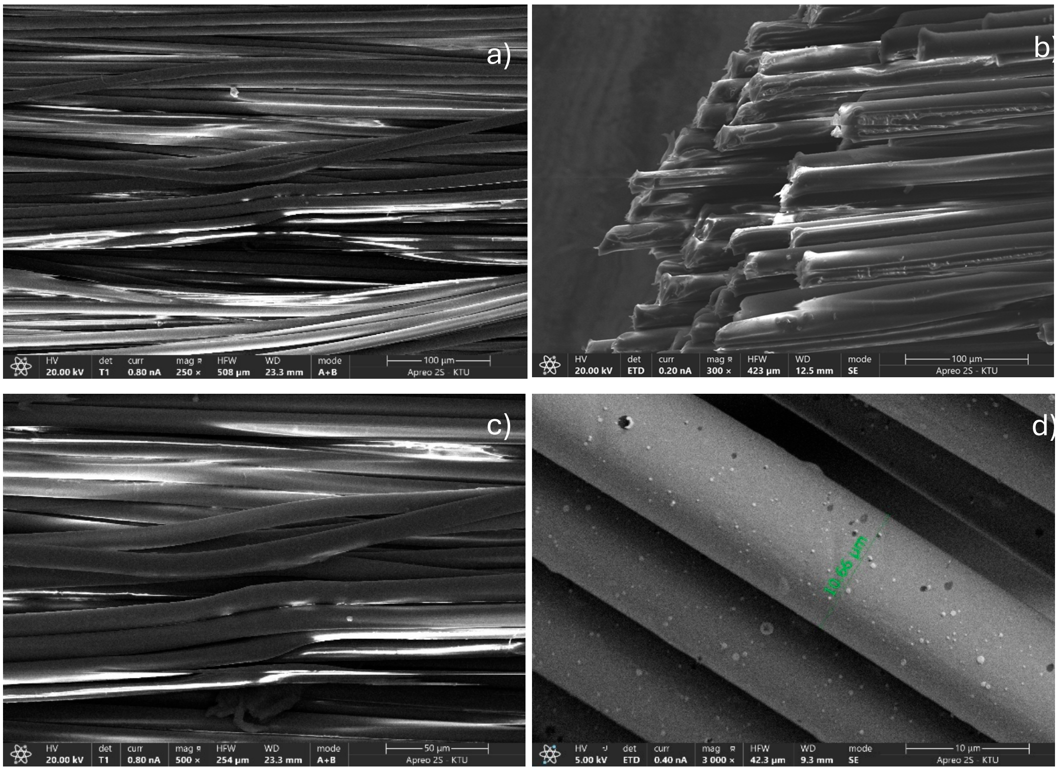


**Figure S6.** SEM images of the PAN fiber at different magnifications: (a) 250×, (b) 300×, (c) 500×, and (d) 3,000×.

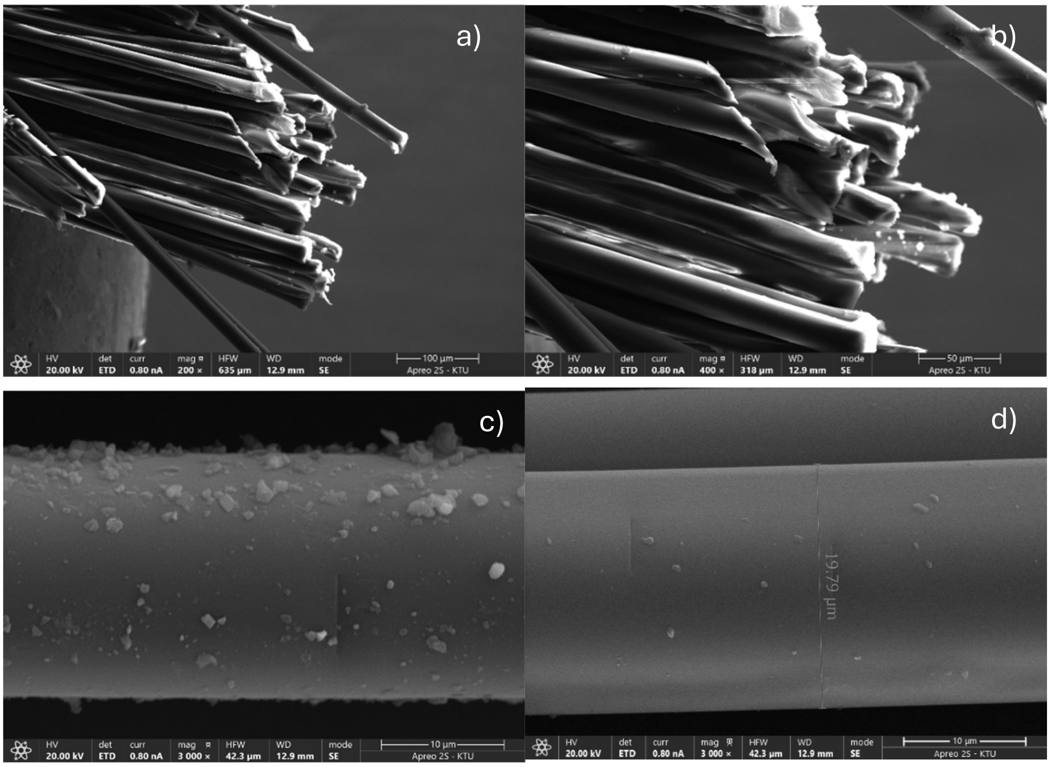


**Figure S7.** SEM images of the PET fiber: (a) 200×, (b) 400×, (c) 3,000×, and (d) fiber diameter.

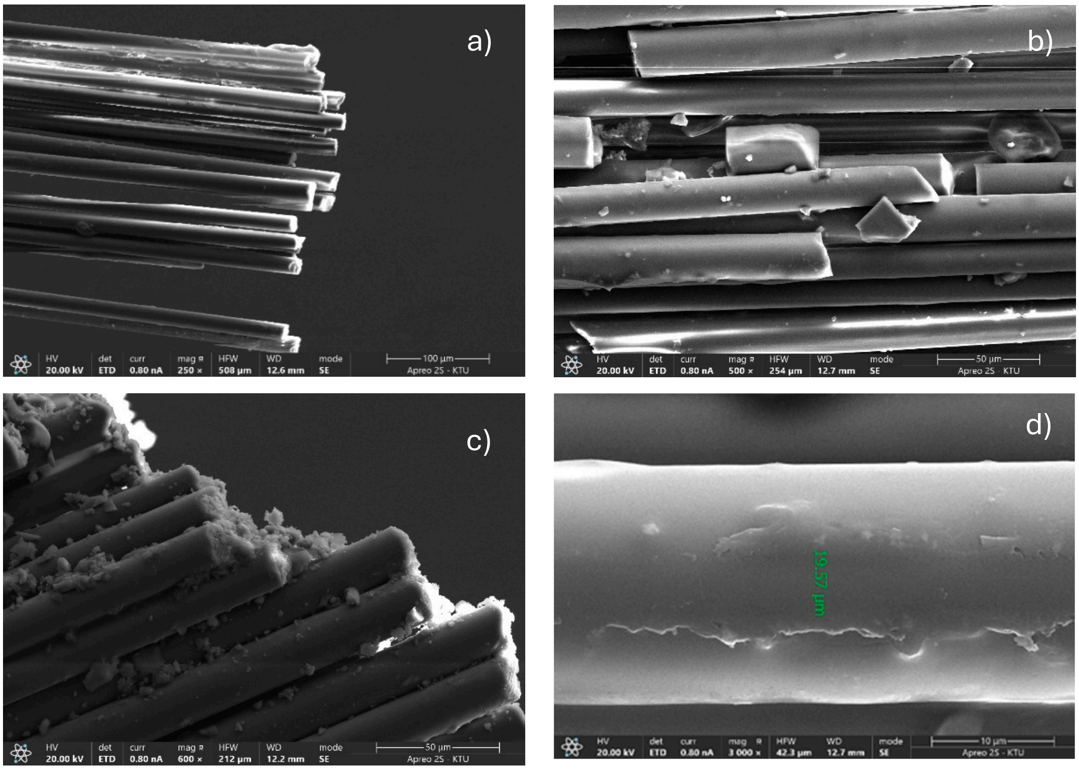


**Figure S8.** SEM images of the basalt fiber at different magnifications: (a) 250×, (b) 500×, (c) 600×, and (d) 3,000×.

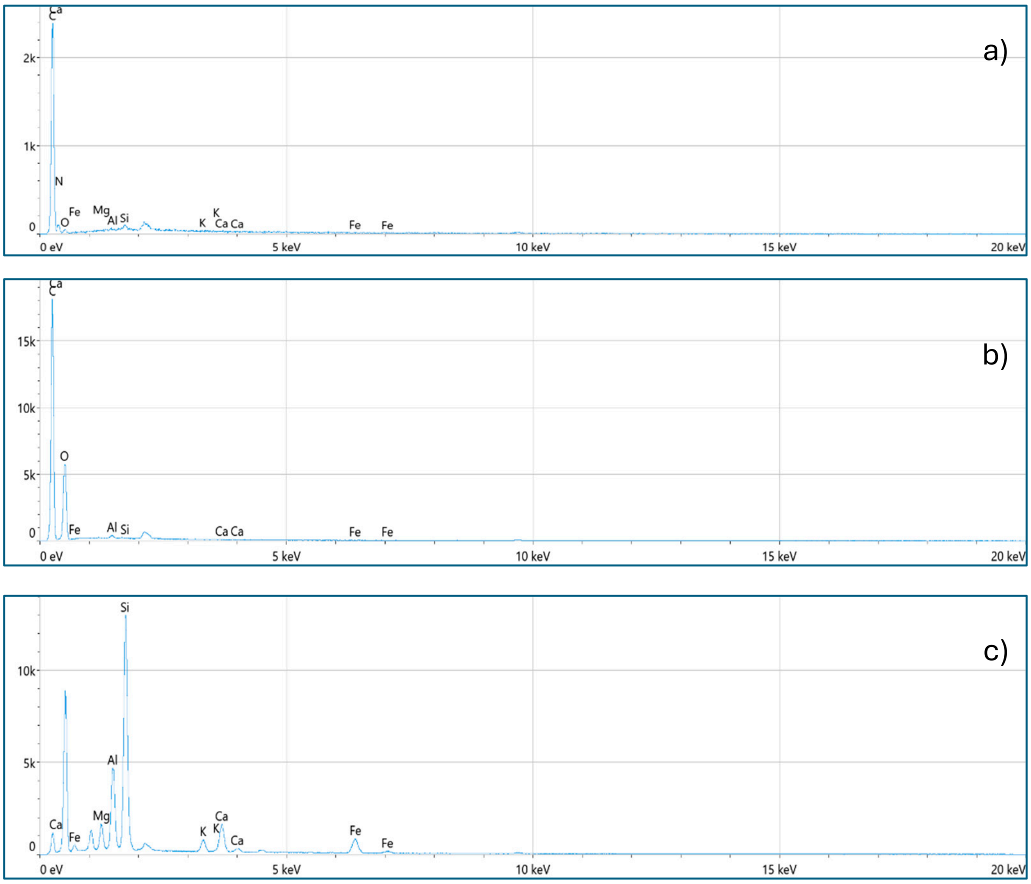


**Figure S9.** EDS spectra of the microfibers: (a) PAN, (b) PET, and (c) basalt fibers.

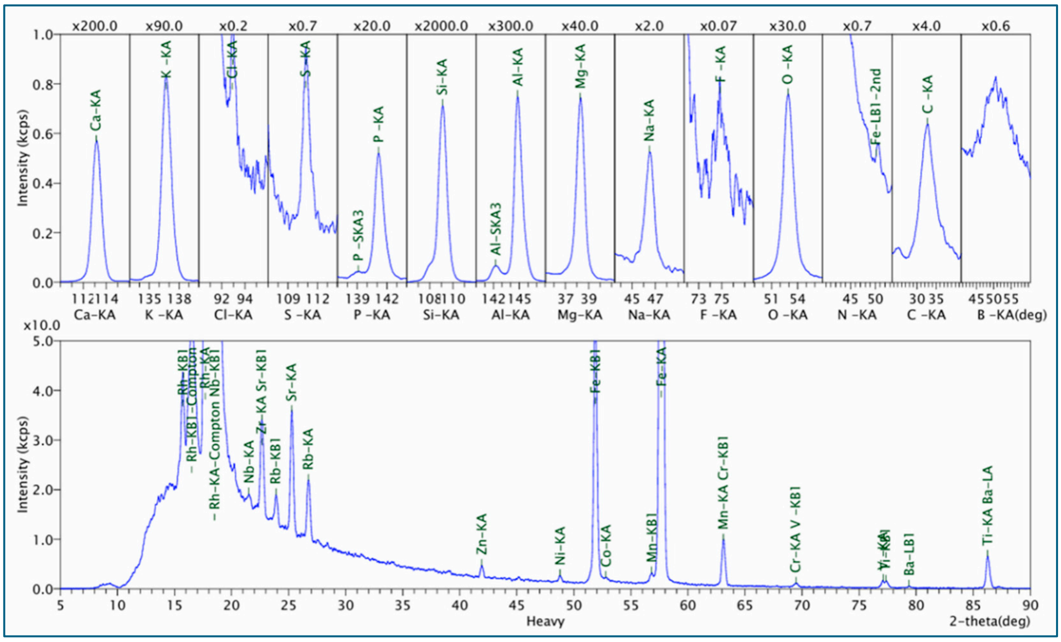


(a)

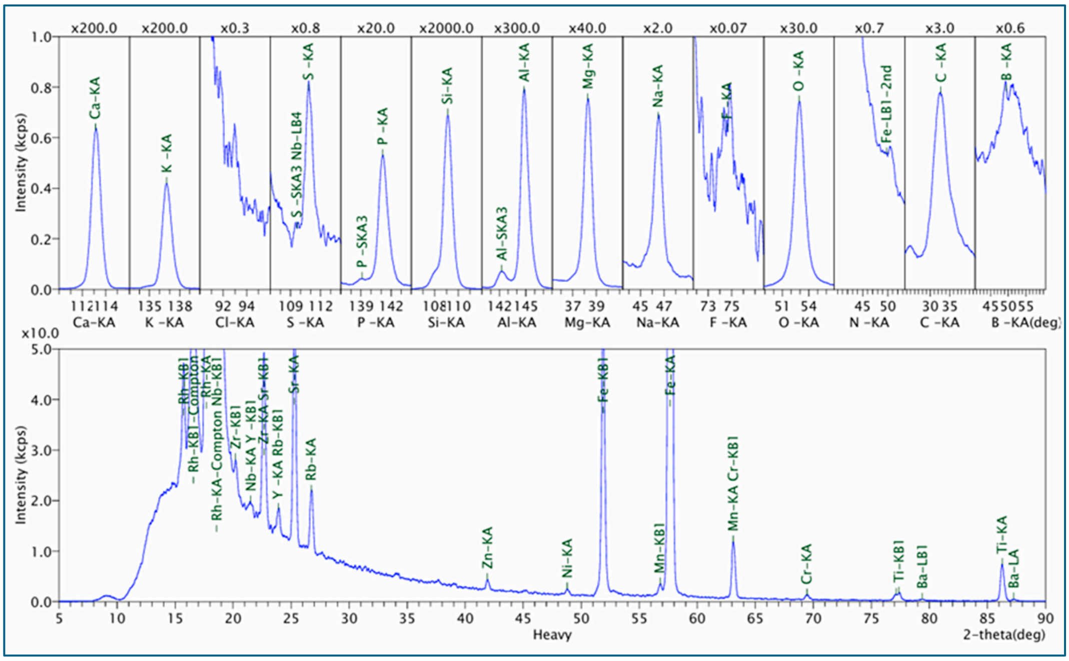


(b)

**Figure S10.** XRF spectra of the diatomites: (a) D1 and (b) D2.

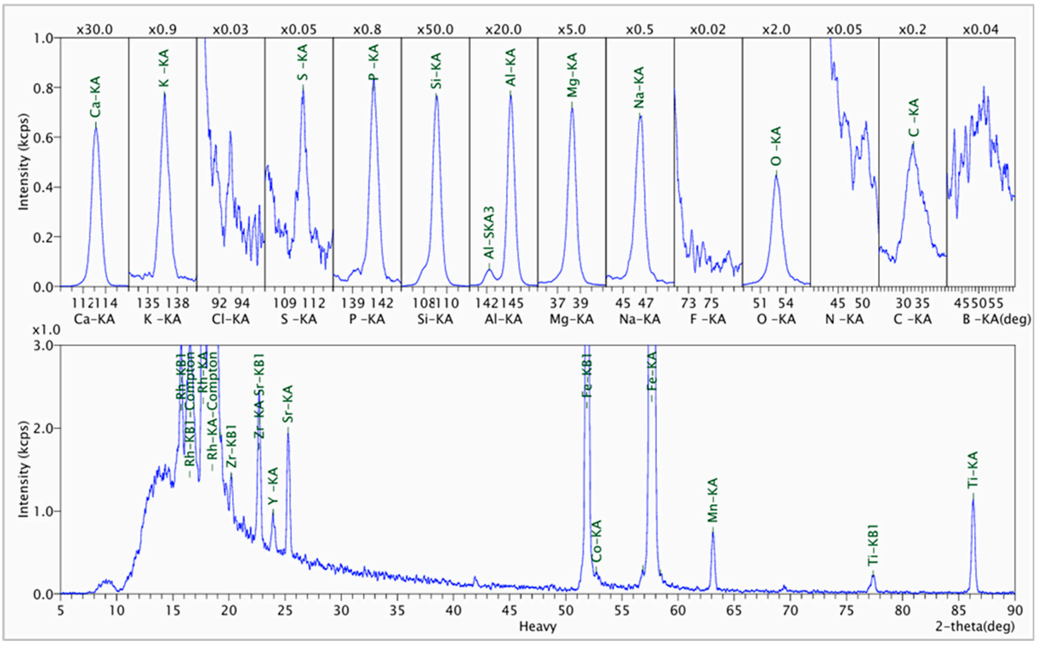


**Figure S11.** XRF spectrum of the basalt filler.

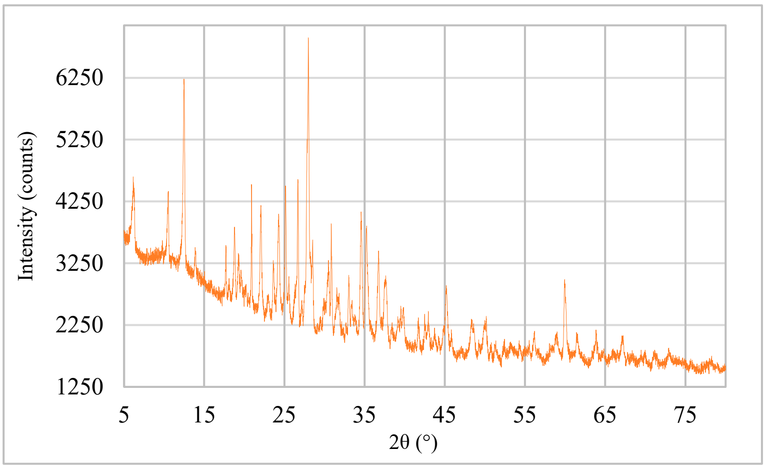


**Figure S12.** XRD pattern of the basalt filler.

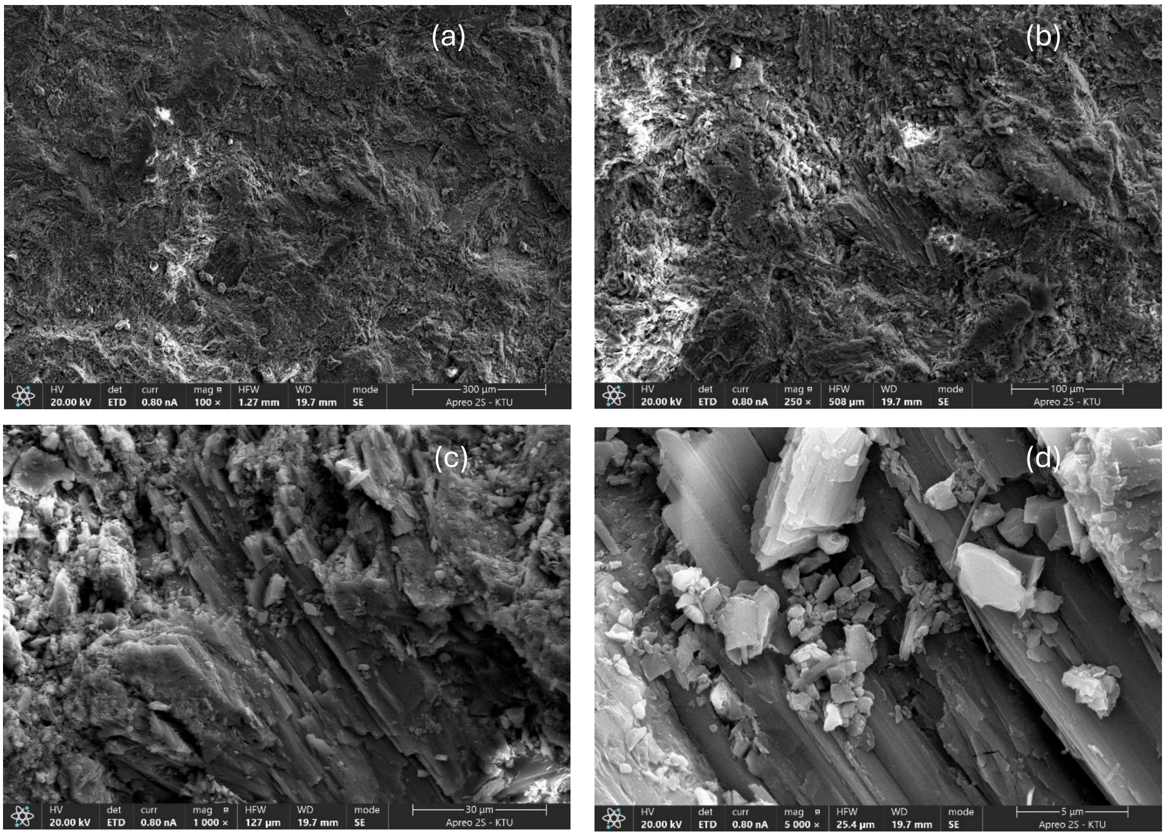


**Figure S13.** SEM images of the 15%D1-PAN fiber-reinforced asphalt mixture at different magnifications: (a) 100×, (b) 250×, (c) 1,000×, and (d) 5,000×.

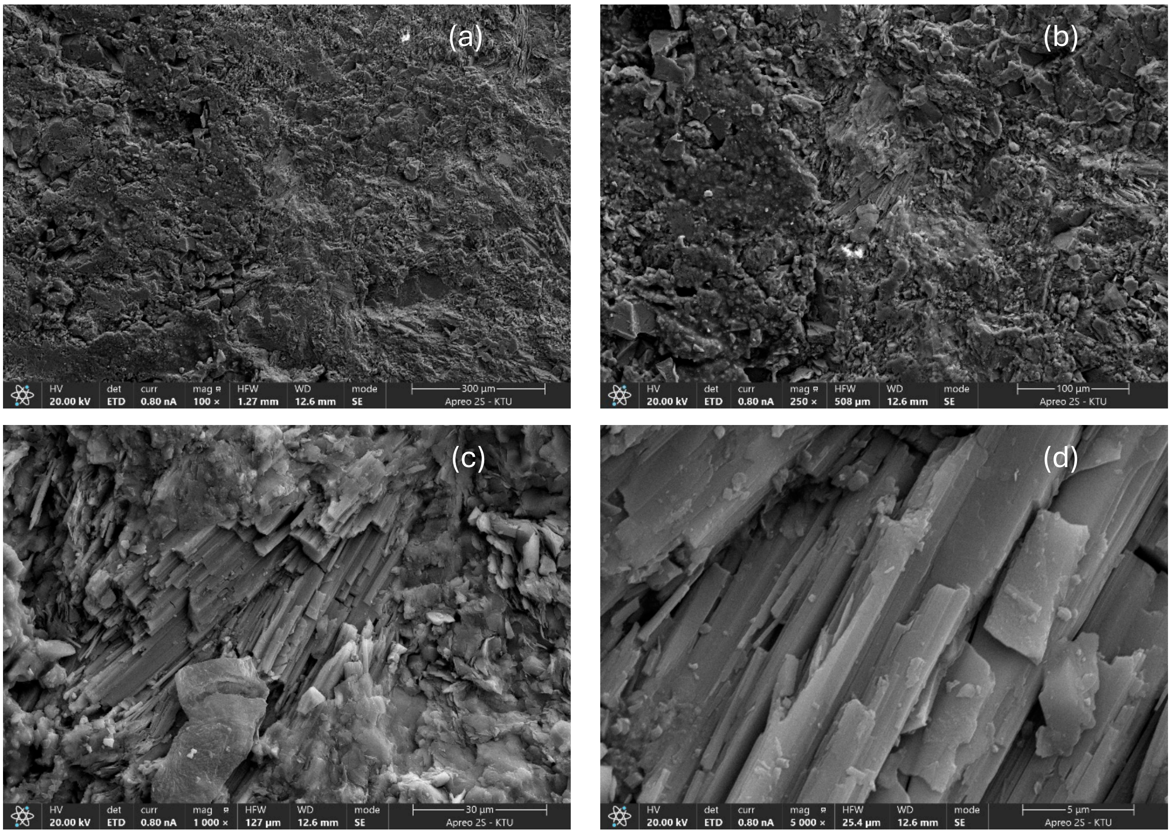


**Figure S14.** SEM images of the 15%D1-PET fiber-reinforced asphalt mixture at different magnifications: (a) 100×, (b) 250×, (c) 1,000×, and (d) 5,000×.

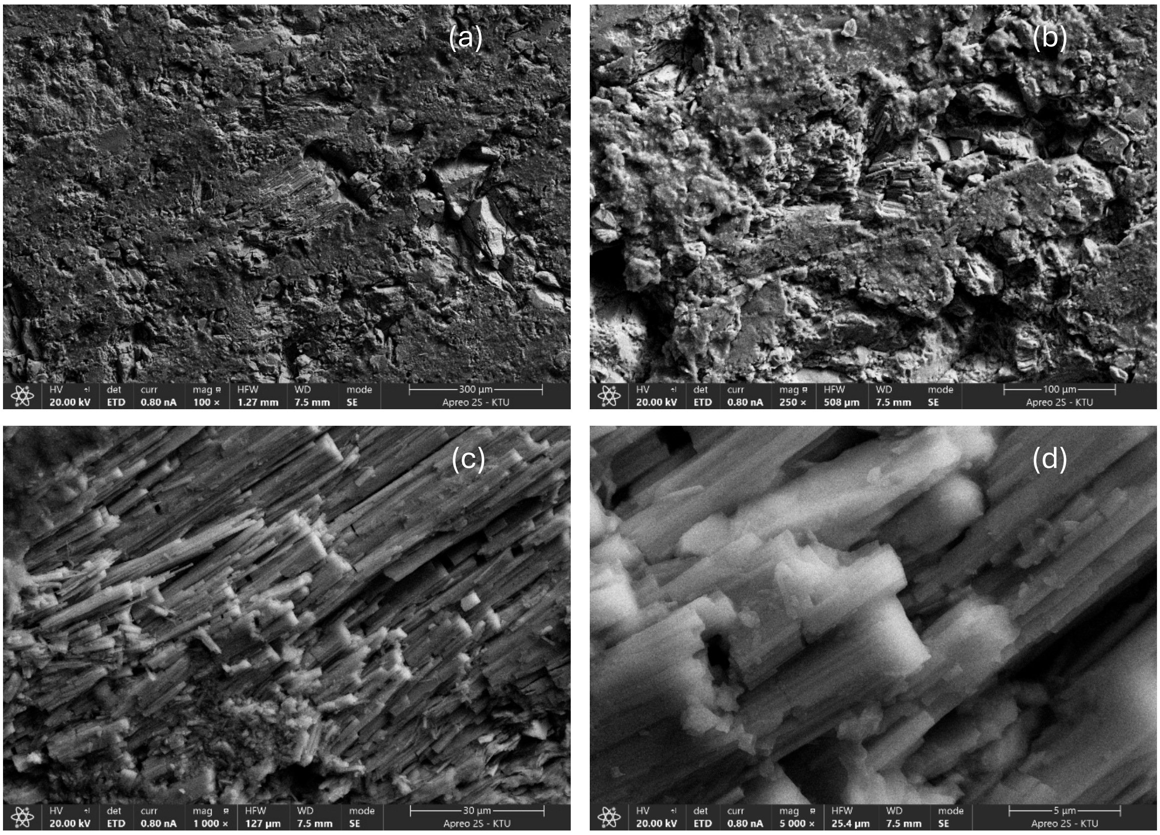


**Figure S15.** SEM images of the 15%D1-BF fiber-reinforced asphalt mixture at different magnifications: (a) 100×, (b) 250×, (c) 1,000×, and (d) 5,000×.

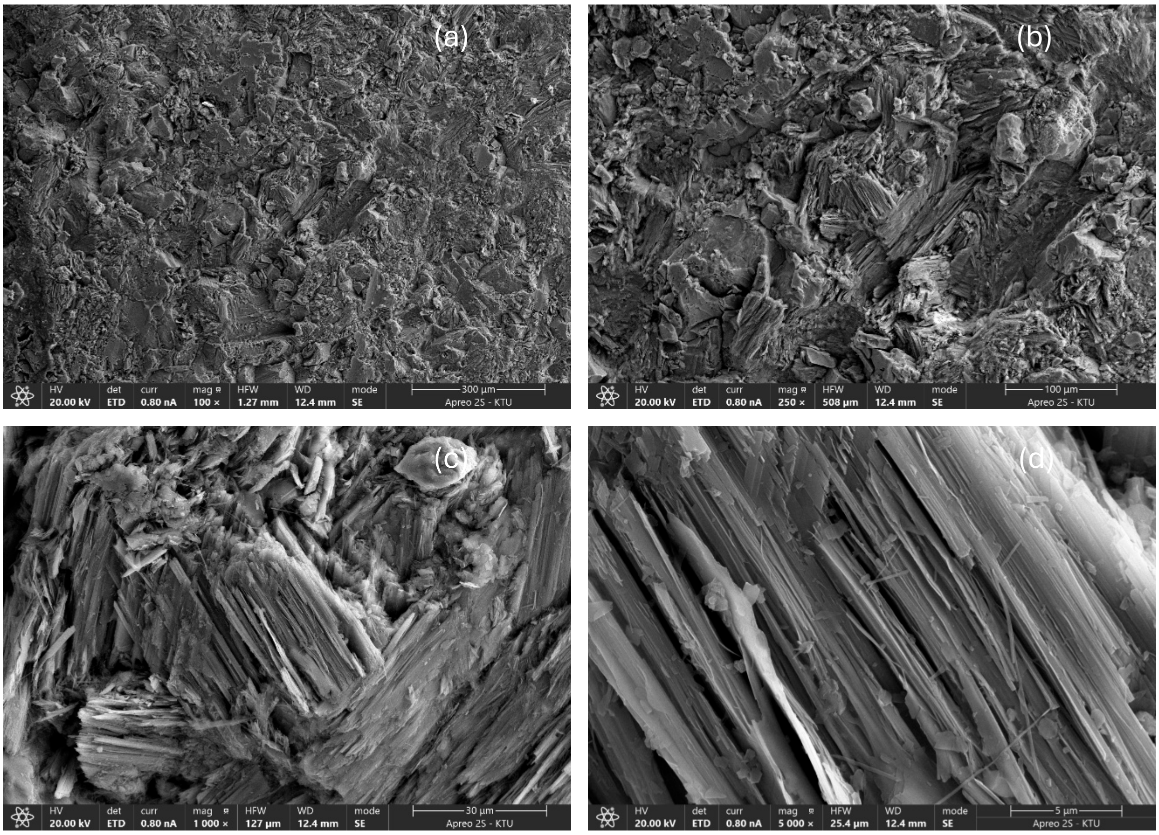


**Figure S16.** SEM images of the 10%D2-PAN fiber-reinforced asphalt mixture at different magnifications: (a) 100×, (b) 250×, (c) 1,000×, and (d) 5,000×.

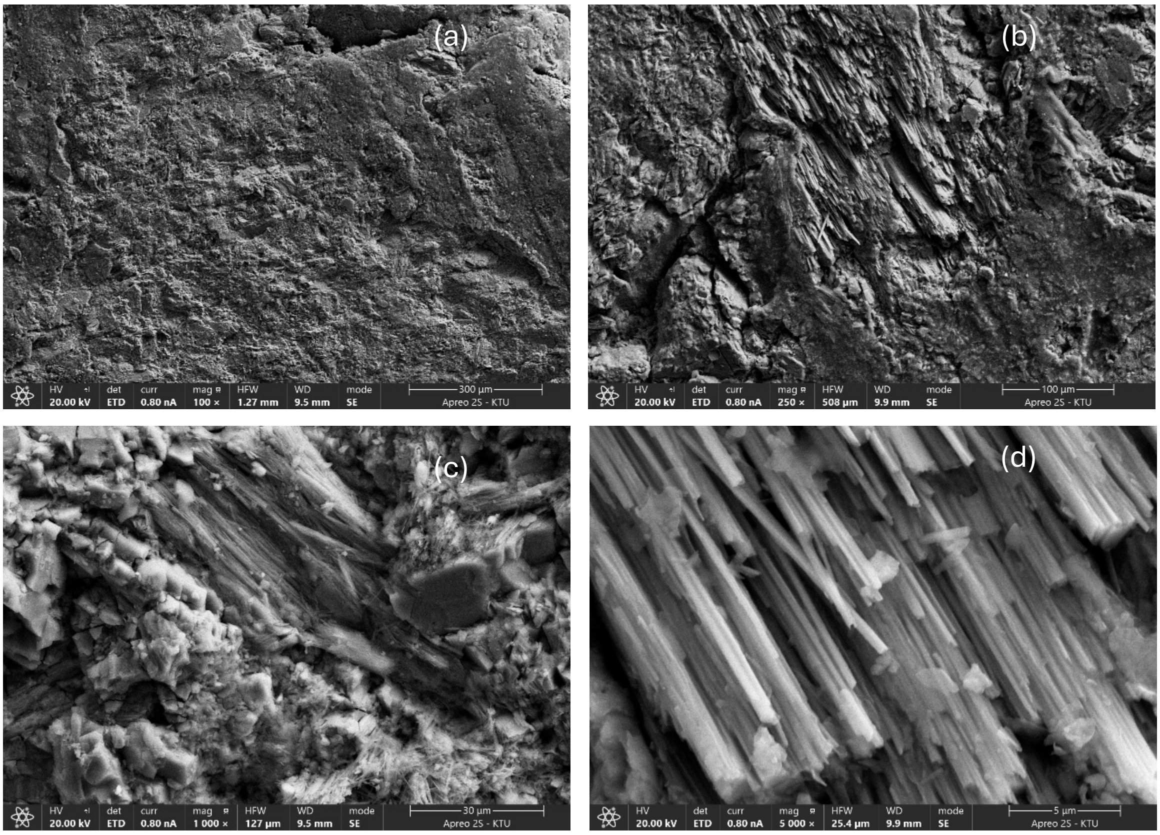


**Figure S17.** SEM images of the 10%D2-PET fiber-reinforced asphalt mixture at different magnifications: (a) 100×, (b) 250×, (c) 1,000×, and (d) 5,000×.

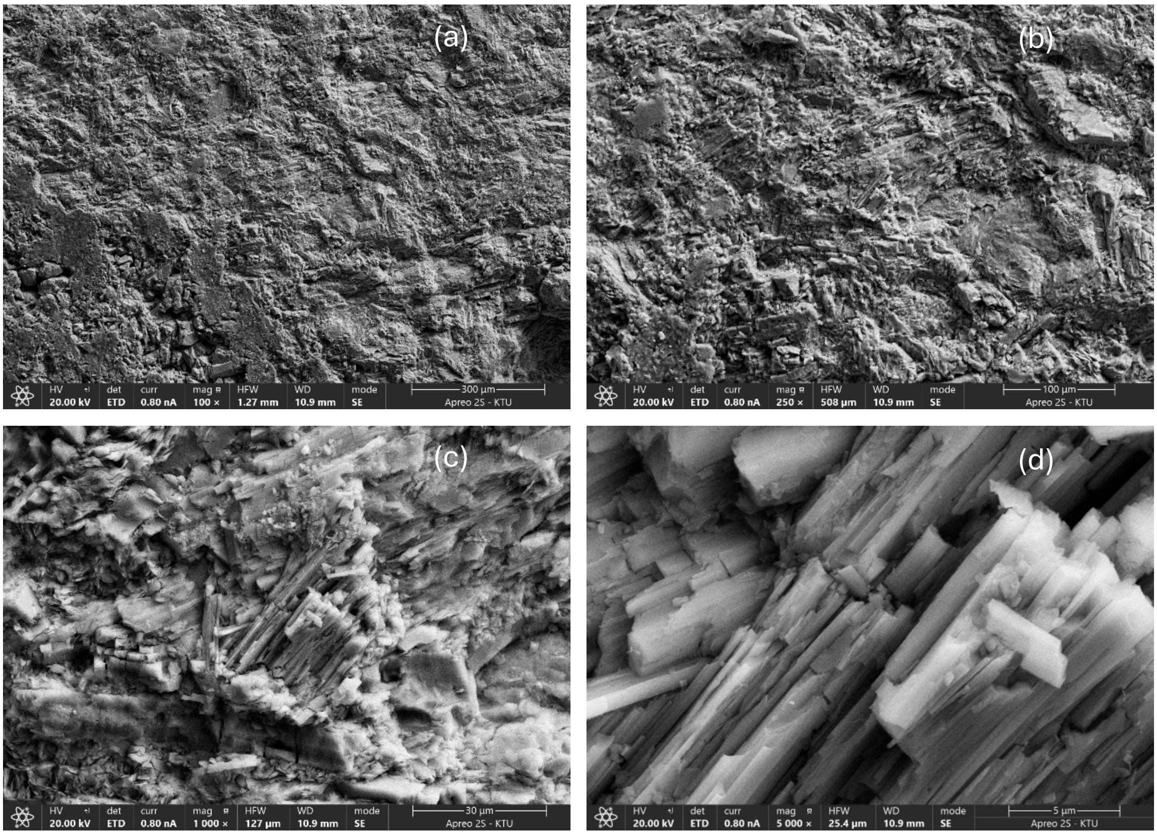


**Figure S18.** SEM images of the 10%D2-BF fiber-reinforced asphalt mixture at different magnifications: (a) 100×, (b) 250×, (c) 1,000×, and (d) 5,000×.

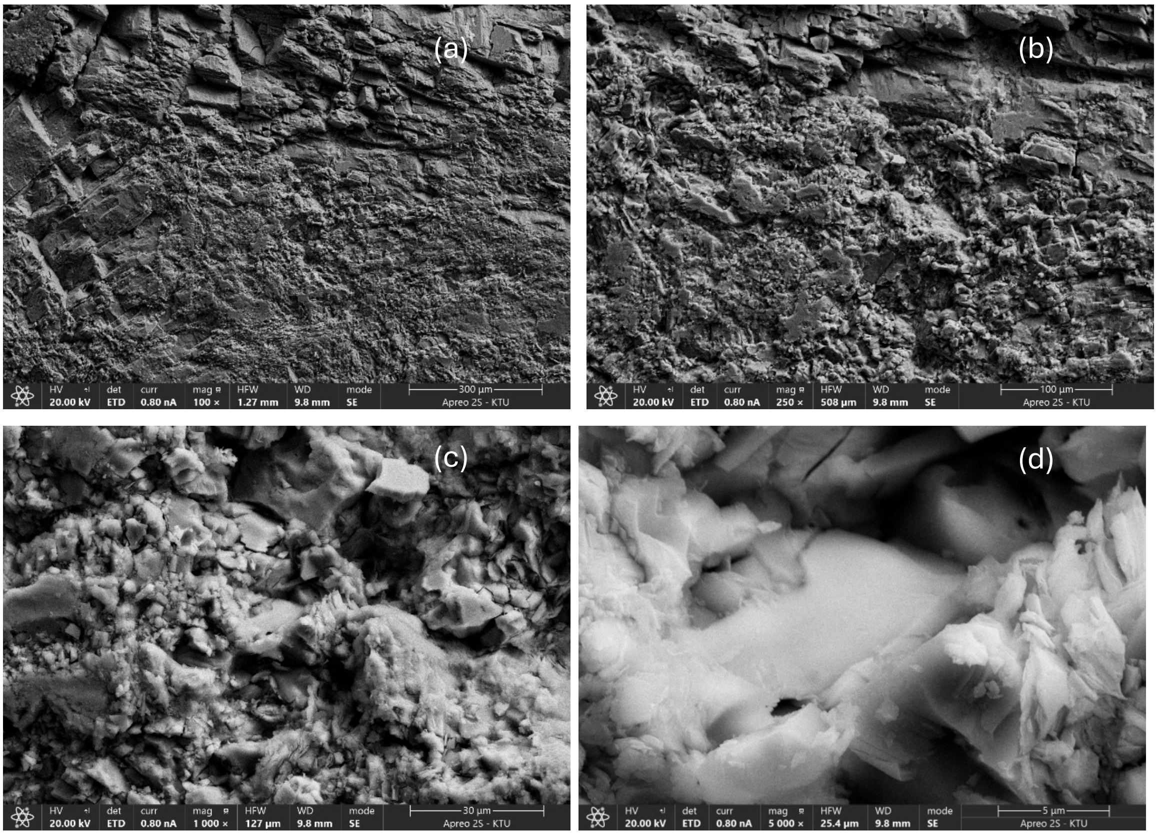


**Figure S19.** SEM images of the unmodified control asphalt mixture at different magnifications: (a) 100×, (b) 250×, (c) 1,000×, and (d) 5,000×.

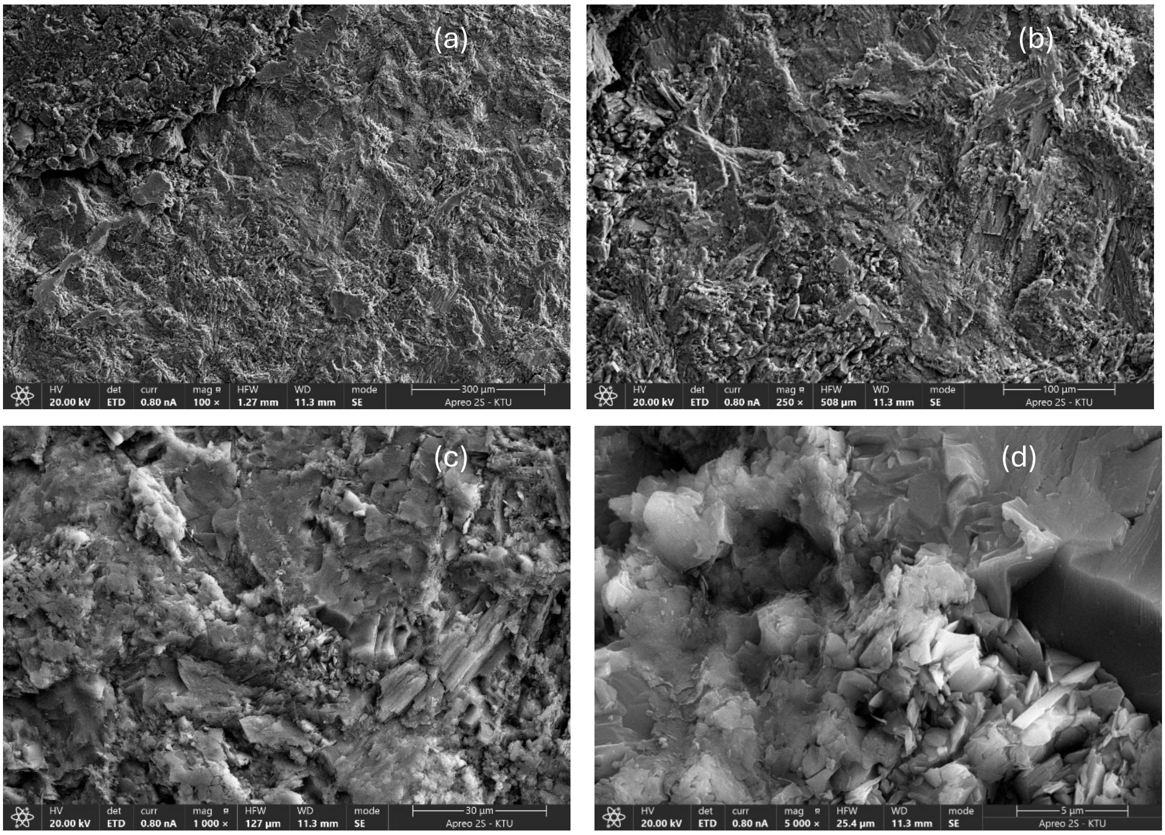


**Figure S20.** SEM images of the 5%SBS-modified asphalt mixture at different magnifications: (a) 100×, (b) 250×, (c) 1,000×, and (d) 5,000×.

**Table S7.** Individual indirect tensile strength (ITS) measurements of unconditioned and moisture-conditioned asphalt mixtures, with group means, sample standard deviations, and tensile strength ratios (TSR). ITS values are expressed in MPa (n = 3 per conditioning group).

| **Mixture** | **$U_1$** | **$U_2$** | **$U_3$** | **$U_{mean}$±SD** | $C_1$ | $C_2$ | $C_3$ | **$C_{mean}$±SD** | **TSR(%)** |
|---|---|---|---|---|---|---|---|---|---|
| Control | 1.403 | 1.566 | 1.444 | 1.471±0.085 | 1.037 | 1.011 | 1.143 | 1.064±0.070 | 72.30 |
| 5%SBS | 1.505 | 1.598 | 1.531 | 1.545±0.048 | 1.244 | 1.212 | 1.304 | 1.253±0.047 | 81.13 |
| 15%D1-PAN | 1.546 | 1.672 | 1.634 | 1.617±0.065 | 1.346 | 1.079 | 1.057 | 1.161±0.161 | 71.76 |
| 15%D1-PET | 1.559 | 1.581 | 1.670 | 1.603±0.059 | 1.335 | 1.075 | 1.054 | 1.155±0.157 | 72.05 |
| 15%D1-BF | 1.586 | 1.633 | 1.724 | 1.648±0.070 | 1.217 | 1.222 | 1.137 | 1.192±0.048 | 72.35 |
| 10%D2-PAN | 1.622 | 1.593 | 1.680 | 1.632±0.044 | 1.410 | 1.329 | 1.312 | 1.350±0.052 | 82.76 |
| 10%D2-PET | 1.605 | 1.574 | 1.637 | 1.605±0.032 | 1.377 | 1.274 | 1.327 | 1.326±0.051 | 82.60 |
| 10%D2-BF | 1.567 | 1.578 | 1.649 | 1.598±0.044 | 1.223 | 1.158 | 1.099 | 1.160±0.062 | 72.58 |

Note: U: unconditioned; C: moisture-conditioned; SD: sample standard deviation. TSR was calculated as the ratio of the mean conditioned ITS to the mean unconditioned ITS, multiplied by 100. Each group comprised three independent specimens.
